# Supplementary material for: MS-Based Approaches Enable the Structural Characterization of Transcription Factor/DNA Response Element Complex
Source: Biomolecules. 2019 Sep 26;9(10):535. doi: 10.3390/biom9100535 (PMC6843354; doi:10.3390/biom9100535)
Supplement: Supplementary file 1 [file biomolecules-09-00535-s001.pdf]

biomolecules

Supplementary Materials

MS-Based Approaches Enable the Structural Characterization of Transcription Factor/DNA Response Element Complex

Lukáš Slavata [1,2], Josef Chmelík [1,2], Daniel Kavan [1,2], Růžena Filandrová [1,2], Jan Fiala [1,2], Michal Rosůlek [1,2], Hynek Mrázek [1], Zdeněk Kukačka [1], Karel Vališ [1], Petr Man [1,2], Michael Miller [3], William McIntyre [3], Daniele Fabris [3] and Petr Novák [1,2,*]

[1] Institute of Microbiology, The Czech Academy of Sciences, Prague 14220, Czech Republic; lukas.slavata@gmail.com (L.S.); chmelik@biomed.cas.cz (J.C.); kuan@maradan.org (D.K.); ruzena.liskova@gmail.com (R.F.); FiJi.cs@seznam.cz (J.F.); rosulek.michal@gmail.com (M.R.); hynek.mrazek@gmail.com (H.M.); zdenek.kukacka@biomed.cas.cz (Z.K.); karel.valis@biomed.cas.cz (K.V.); pman@biomed.cas.cz (P.M.)

[2] Faculty of Science, Charles University, Prague 12843, Czech Republic

[3] RNA Institute, University at Albany, State University of New York, Albany, NY 12222, USA; mrmiller@albany.edu (M.M.); wdmcintyre@albany.edu (W.M.); dfabris@albany.edu (D.F.)

* Correspondence: pnovak@biomed.cas.cz; Tel.: +42-0325-873-610

Received: 19 July 2019; Accepted: 24 September 2019; Published: date

1. Supplementary methods

1.1. *Protein recombinant expression and purification*

Full length DBD of FOXO4 (residues 82–207) was recombinantly produced as a N-terminal His-tag fusion protein in BL21 competent *E. coli* (DE3). The pET-15b plasmid carrying His-tag, thrombin cleavage site and FOXO4-DBD sequences was used as a vector for transformation. After initial growth at 37°C, protein expression in transformed cells was induced by addition of IPTG to the cell culture cooled to 30°C and performed for 12 h. Subsequent affinity purification was performed on a column filled with a TALON Superflow Resin (Clontech Laboratories, USA) charged with Co^{2+} according to the manufacturer protocol. Cleavage of His-tag was accomplished overnight at 4°C by treatment with human thrombin (2U per mg of recombinant protein). Removal of thrombin, cleaved His-tag and buffer exchange were carried out by gel permeation chromatography on a ENrich SEC 70 10 × 300 column (Bio-Rad Laboratories, USA).

1.2. Electrospray ionization (ESI) Fourier transform ion cyclotron (FTICR) MS analysis

ESI-FTICR MS analysis was carried out to verify the identity of recombinant protein samples and the integrity of the FOXO4-DBD•DBE complex. This type analysis was repeated before and after cross-linking reactions to monitor the outcome and verify the composition of intact samples before protease digestion. Samples were typically concentrated by ultrafiltration on Amicon Ultra 0.5 mL centrifugal filters (Merck, USA) and then diluted to a final 10μM protein concentration in either a 7.5mM AA, 2% AcOH solution that preserved complex association, or a 7.5mM AA, 1% AcOH, 50% MeOH solution to induce complex denaturation. Each sample was introduced into the ESI source by using a syringe pump operating at a 2 μL/min flow rate. The analysis was carried out on a Bruker Daltonics (Billerica, USA) 15T-solariX XR FTICR mass spectrometer, which was calibrated by using a solution of sodium trifluoroacetate (NaTFA) to achieve a typical 1 ppm mass accuracy. Mass spectra were acquired in positive mode over the 250 - 4000 m/z range for 3 min. DataAnalysis 4.2 was employed to accomplish data interpretation and processing.

1.3. Hydrogen-deuterium exchange

1.3.1. HDX reaction

FOXO4-DBD (20μM) or FOXO4-DBD/DBE complex (20μM, 1:1 molar ratio) were pre-incubated for 1 h at 20°C in an H_2O-based buffer at pH 7.4 consisting of 10mM HEPES and 50mM NaCl. Deuterium exchange was initiated by 10-fold dilution of such samples into an equivalent D_2O-based buffer (10mM HEPES, 50mM NaCl, pD 7.4). The reaction was allowed to proceed at 20°C and quenched at pre-determined intervals (i.e., 0.33, 2, 5, 10, 30, 60, 180, 300 min) by removing aliquots containing 100 pmol of protein and mixing them immediately with a 1M glycine/HCl buffer at pH 2.35, followed by rapid freezing in liquid nitrogen. All HDX experiments were performed as triplicate.

1.3.2. UHPLC/ESI-FTICR MS analysis.

Samples were stored at -80°C and thawed immediately before LC-MS analysis. Each sample was injected into a home-built LC system based on a LC-20AD pump (Shimadzu corp., JPN), which was maintained at 0°C to minimize hydrogen back-exchange. Protein digestion was carried out in column(s) with immobilized protease(s) at a flow rate of 100 μL/min of 0.4% formic acid (FA) in water. The ensuing peptide products were trapped and desalted on a C8 reversed-phase microtrap (Michrom BioResources, USA) for 3 min under the same flow rate and solvent composition. Trapped peptides were eluted at a constant flow rate of 15 μL/min by using a 1200 HPLC pump (Agilent Technologies, USA), which provided a linear 5 min gradient from 5-35% of solution B (0.4% FA, 95% MeCN in water), followed by a quick step to 99% B. The released peptides were finally separated on a reversed-phase analytical column (Jupiter C18, 0.5 × 5 mm, 5 μm, 300 Å, Phenomenex, USA).

Solution A consists of 0.4% FA, 2% MeCN in water. The same protocol was used to accomplish the peptide mapping of non-deuterated samples, except that H_2O-based buffer was used instead of the D_2O-based equivalent and that 300 pmol of protein was injected into the LC system. Separated peptides were introduced into the ESI source of a Bruker Daltonics (Billerica, USA) 9.4T Apex Ultra Qe FTICR mass spectrometer. Peptide mapping was carried out in positive ion mode by performing data-dependent broadband analysis. In this experiment, each MS scan was followed by six MS/MS scans, in which the six most abundant peptide ions detected in the MS scan were submitted to collision-induced dissociation.

1.3.3. Data processing.

The MS/MS data acquired in the 300 - 1800 m/z range were processed by DataAnalysis 4.2 (Bruker Daltonics, USA) and then searched by MASCOT 2.2 search engine against a library of possible digestion products obtained from the FOXO4-DBD sequence. The hits were further processed by using the MSTools package [1]. Deuteration rate was determined in the course of positive broadband MS mode analysis (300 - 1800 m/z range) and the ensuing data were processed by using the home-built DeutEx software (unpublished). For each product, the relative deuteration rate was determined as a percentage of maximum possible number of exchangeable amide hydrogens in each peptide, which was then recalculated for each amino acid residue as already described in reference [2].

1.4. Quantitative protein-protein cross-linking

1.4.1. Cross-linking reaction

FOXO4-DBD (20μM) or FOXO4-DBD/DBE complex (1: 1 molar ratio) in a buffer solution at pH 7.4 (10mM HEPES, 50mM NaCl) were pre-incubated for 1 h at 20°C and then mixed with either DSG or DSS cross-linking reagents. Unbound FOXO4-DBD samples were mixed with the non-labelled cross-linkers (DSGd0 or DSSd0), whereas bound FOXO4-DBD•DBE complex was mixed with the deuterium-labelled cross-linkers (DSGd4 or DSSd4). All cross-linkers were dissolved in dimethyl sulfoxide (DMSO) to a 6.74mM concentration. The amount of cross-linkers added to each sample provided a 10-fold molar excess over the protein and the addition resulted in a final 5% content of DMSO and 20μM protein concentration in the reaction mixture. After 2 h of cross-linking reaction, corresponding unlabelled and deuterium-labelled samples (e.g., DSSd0- and DSSd4-traeted) were mixed in 1:1 ratio to enable direct quantification. Control samples obtained by adding pure DMSO in the absence of cross-linker, as well the samples treated with 1:1 mixtures of cross-linkers (e.g., DSGd0/DSGd4 or DSSd0/DSSd4) were prepared at the same time and handled in the same manner. All cross-linking reactions were performed as triplicate. Reaction samples were analyzed by SDS-PAGE to monitor the formation of higher oligomeric structures as a result of cross-linking. This analysis was repeated after trypsin digestion to verify the formation of peptide products to be submitted to LC-MS.

1.4.2. Proteolytic digestion

The pH of samples was adjusted to 8.3 by addition of 100mM ethylmorpholine with 20% of MeCN. Next, trypsin dissolved in water was added to reach a final 1:20 (trypsin:protein) weight ratio. Digestion was performed overnight at 37°C and quenched by adjusting the solution to an acidic pH with 1% FA.

1.4.3. HPLC/ESI-FTICR MS analysis

Peptide samples diluted in 0.1% FA to a final 0.05 μg/μL concentration were injected (0.1 μg per injection) onto a trap column (ZORBAX 300SB-C18, 5 μm, 0.30 x 5 mm cartridge, Agilent, USA), in which the products were desalted by a 10 μL/min flow of 0.1% FA in water for 3 min (LC-20AD HPLC pump, Shimadzu corp., JPN). Desalted peptides were eluted from the trap column at a

constant flow rate of 0.5 μL/min (HPLC pump 1200, Agilent Technologies, USA) by a two-step linear gradient. The program consisted of a slow ramp from 2 to 45% of solution B (0.1% FA, 98% MeCN in water) in 43 min, followed by a fast step from 45 to 95% in 3 min. Solution A consisted of 0.1% FA and 2% MeCN in water. The peptides were then separated on-line on an analytical column (ZORBAX 300SB-C18, 0.3 x 150 mm, 3.5 μm, Agilent, USA). Finally, the peptide products were introduced into the ESI source of a Bruker Daltonics (Billerica, USA) 15T-solarix XR FTICR mass spectrometer. Mass spectral data was acquired in positive mode across a 250 - 2000 m/z range.

1.4.4. Data processing and quantification

MS data were processed by DataAnalysis 4.2 (Bruker Daltonics, USA). In particular, the SNAP 2.0 algorithm was used to generate deconvoluted spectra and corresponding lists of singly-charged monoisotopic masses. Data obtained from control and cross-linked samples were compared with theoretical libraries by using the home-built LinX software (available online) to identify the sough-after cross-linking products. All spectra containing the signal of identified cross-links were summed and further processed by mMass 5.4.1 [3]. For each cross-linking product, the ratio between light (d0) and heavy (d4) cross-linker incorporation was calculated by using the envelope fit tool, which operated by generating theoretical profiles and then fitting them to the experimental data according to linear combination and least-square fitting. Control samples were also checked for possible 1:1 ratios of light and heavy forms.

1.5. Protein-DNA chemical cross-linking

1.5.1. Transplatin reaction

Substrate solutions containing 25μM of either FOXO4-DBD or FOXO4-DBD•DBE complex in 150mM ammonium acetate (AA) at pH 6.85 and reagent solutions containing 1mM trans-platinum(II)diammine dichloride (transplatin) prepared by dilution of 20mM stock in dimethylformamide (DMF) were pre-incubated separately for 1 h at 18°C. Aliquots were then mixed to obtain final concentrations of 20μM substrate and 200μM reagent in 150mM AA and 1% DMF. The reaction was carried out for 14 h at 18°C. Control samples containing the same amount of DMF in water (no transplatin) were prepared at the same time and handled in the same manner. Reaction and control samples were analyzed by native and denaturing DNA polyacrylamide gel electrophoresis, SDS polyacrylamide gel electrophoresis, ESI-FTICR MS and HPLC/ESI-FTICR MS.

1.5.2. Complex digestion

The nucleic acid component of the FOXO4-DBD•DBE complex was digested with specific nucleases to facilitate the identification of nucleotides that were directly conjugated with the protein component by the cross-linking reaction. The reaction mixture was added with a solution of Bal-31 nuclease (0.15 U per 1 μg of duplex DNA) in a 40mM TRIS/HCl buffer at pH 8.0, which contained 24mM $MgCl_2$, 24mM $CaCl_2$, and 2mM EDTA. Nuclease digestion was carried out for 1 h at 30°C. The samples were subsequently subjected to trypsin digestion as described above.

1.5.3. HPLC/ESI-FTICR MS analysis

The digestion mixtures were analyzed in both positive and negative ion mode to better capitalize on the charging of products that may have a predominant peptide (positive) or oligonucleotide (negative) character. The former was accomplished as described above for trypsin digests, with only slightly different conditions: 0.375 μg of material per injection; 10 μL/min two-step linear gradient from 5 to 30% in 25 min and from 30 to 95% in 5 min. Mass spectral data were acquired in positive, data-independent mode over the 250 - 2500 m/z range. In this experiment, each MS scan (0.2 s accumulation) was followed by an MS/MS scan (0.8 s accumulation) with a broad isolation window (250 - 2500 m/z) and a fixed 15eV collision-induced dissociation energy. The analysis in negative ion mode was accomplished as described above, but with the following

variations: 0.375 μg of material per injection; 10 mM ammonium bicarbonate at pH 7.5 for trapping and desalting operations; separation accomplished on a ZORBAX 300Extend-C18, 0.3 x 150 mm, 3.5 μm (Agilent, USA) analytical column by using a 10 μL/min two step linear gradient from 10 to 55% in 30 min and from 55 to 99% in 5 min; A solution (4% hexafluoroisopropyl alcohol (HFIP) and 0.1% triethylammonium acetate (TEAA) in water at pH 7.5), B solution (1% hexafluoroisopropyl alcohol (HFIP), 0.025% triethylammonium acetate (TEAA), 75% and MeOH in water at pH 7.5). Mass spectral data were acquired in negative, data-independent mode over the 200 - 2500 m/z range. Also, in this experiment, each MS scan (0.2 s accumulation) was followed by an MS/MS scan (0.8 s accumulation) with a broad isolation window (250 - 2500 m/z) and a fixed 15eV collision-induced dissociation energy.

1.5.4. Data interpretation

MS and MS/MS data were processed by using DataAnalysis 4.2 (Bruker Daltonics, USA). In particular, the SNAP 2.0 algorithm was employed to generate deconvoluted spectra of detected species to be compared to a theoretical library of putative peptide-oligonucleotide conjugates. The process required combining the average content of biogenic isotopes in average peptides with a fictitious additional constant corresponding to $C_{129}H_{160}N_{54}O_{74}P_{12}PtN_2H_4$, which represented a single strand of the DBE component conjugated by the transplatin cross-linker. This expedient ensured the proper determination of monoisotopic masses of putative protein-DNA cross-links products. The lists of monoisotopic masses provided by the MS determinations were searched for possible protein-DNA cross-links by using the home-built LinX software (available online). Further validation of initial hits was achieved by manually comparing the experimental isotopic patterns with the model isotopic patterns suggested by LinX, which were expected to manifest unique features conferred by the phosphate and platinum contributions from the oligonucleotide and cross-linker moieties. The MS/MS data were visually inspected in similar fashion to identify fragmentation products corresponding to the sought-after protein-DNA cross-links.

1.5.4. Molecular modeling

The program Modeller [4] was employed to generate models of FOXO4-DBD and FOXO4-DBD•DBE according to the spatial constraints afforded by the HDX and cross-linking experiments. Initially, six different structures available in the Protein Data Bank were utilized as possible templates to guide homology modeling, which displayed different levels of sequence identity (s. i.) with our target. For instance, the 1E17 (100% s. i.), 2K86 (83% s. i.), and 2KIU (51% s. i.) structures contain only the apo-protein with no bound DNA, whereas 3L2C (100% s. i.), 2UZK (83% s. i.), and 2A07 (48% s. i.) include also the latter. These structures, however, covered only the 101-176 residues of the DBD sequence, whereas our intended target covered the 82-207 section to include the additional flanking sequences that had eluded structural elucidation. The missing regions spanning G_{74}-Q_{100} at the N-terminus and N_{177}-A_{207} at the C-terminus of the DBD sequence were generated directly in Modeller by using distance restraints derived from our XL data. The online server *make-na* (http://structure.usc.edu/make-na/) was employed to generate the initial structure of DBE duplex in an ideal B-DNA conformation.

The types of cross-linking restraints informing these operations included C^{α} - C^{α} distances that were set to 20.5 ± 3.0 Å for DSG and 24.2 ± 3.0 Å for DSS, respectively. The maximal LYS C^{α}- LYS C^{α} cross-link distance was calculated as a sum of the spacer arm length [5] and the distances between C^{α} and N^{ζ} atoms (for both LYS involved in the cross-link) where all χ side-chain torsion angles are set to the trans conformation. The same approach was used for the calculation of the maximal C^{α}-C^{α} cross-link distances for other amino acids. For cross-links involving the N-terminal residue, the N_{term} - C^{α} distance was set to 14.1 ± 3.0 Å for DSG and 17.8 ± 3.0 Å for DSS, respectively. A distance of 5.5 ± 2.5 Å [6,7] was assigned between atoms bridged by the transplatin reagent. For all distance restraints, a 3-Å standard deviation was used to account for the intrinsic flexibility of linker spacers and side chains involved in the conjugate. The initial structures were submitted to limited molecular dynamics (MD) simulations in the Modeler package to eliminate any possible angle strains and

steric clashes. Subsequently, this process a simulated annealing protocol in the torsion angle space was accomplished in CNS [8]. An ensemble of 50 structures was calculated for each starting model. During simulations, the coordinates afforded by the initial PDB templates and DBE structure, as well as the XL distances, were kept fixed. The resulting models were visualized by using Pymol [9].

The HADDOCK [10] program was utilized to perform docking experiments between FOXO4-DBD and DBE substrate. The process designated the D_{139}-L_{154} stretch as active residues on the basis of the results of HDX experiments. The THY_{16}-ADE_{21} region was designated as active because of its high degree of conservation in the observed consensus sequences. The passive residues were automatically defined around the active ones. The WeNMR/WestLife infrastructure [11] was used to carry out the computationally intensive docking calculations. The first structure of the cluster of the FOXO4-DBD•DBE complex, which displayed the best HADDOCK score for each run, was used for the subsequent modeling operations.

References

1. Kavan, D.; Man, P. MSTools—Web based application for visualization and presentation of HXMS data. *Int. J. Mass Spectrom.* **2011**, *302*, 53–58.
2. Trcka, F.; Durech, M.; Man, P.; Hernychova, L.; Muller, P.; Vojtesek, B. The assembly and intermolecular properties of the Hsp70-Tomm34-Hsp90 molecular chaperone complex. *J. Biol. Chem.* **2014**, *289*, 9887–901.
3. Strohalm, M.; Kavan, D.; Novák, P.; Volný, M.; Havlícek, V. mMass 3: a cross-platform software environment for precise analysis of mass spectrometric data. *Anal. Chem.* **2010**, *82*, 4648–51.
4. Webb, B.; Sali, A. Comparative Protein Structure Modeling Using MODELLER. In *Current Protocols in Bioinformatics*; John Wiley & Sons, Inc.: Hoboken, NJ, USA, 2016; pp. 5.6.1–5.6.37.
5. Pierce Chemicals *Double agents cross-linking reagents selection guide.*; Pierce Chemicals: Rockford, IL, 1999;.
6. Dans, P.D.; Crespo, A.; Estrin, D.A.; Coitiño, E.L. Structural and Energetic Study of Cisplatin and Derivatives: Comparison of the Performance of Density Funtional Theory Implementations. *J. Chem. Theory Comput.* **2008**, *4*, 740–50.
7. Rosen, M.S.; Spokoyny, A.M.; Machan, C.W.; Stern, C.; Sarjeant, A.; Mirkin, C.A. Chelating Effect as a Driving Force for the Selective Formation of Heteroligated Pt(II) Complexes with Bidentate Phosphino-Chalcoether Ligands. *Inorg. Chem.* **2011**, *50*, 1411–1419.
8. Brünger, A.T.; Adams, P.D.; Clore, G.M.; DeLano, W.L.; Gros, P.; Grosse-Kunstleve, R.W.; Jiang, J.S.; Kuszewski, J.; Nilges, M.; Pannu, N.S.; et al. Crystallography & NMR system: A new software suite for macromolecular structure determination. *Acta Crystallogr. D. Biol. Crystallogr.* **1998**, *54*, 905–21.
9. W.L. DeLano The PyMOL Molecular Graphics System 2002.
10. Dominguez, C.; Boelens, R.; Bonvin, A.M.J.J. HADDOCK: A Protein−Protein Docking Approach Based on Biochemical or Biophysical Information. *J. Am. Chem. Soc.* **2003**, *125*, 1731–1737.
11. Wassenaar, T.A.; van Dijk, M.; Loureiro-Ferreira, N.; van der Schot, G.; de Vries, S.J.; Schmitz, C.; van der Zwan, J.; Boelens, R.; Giachetti, A.; Ferella, L.; et al. WeNMR: Structural Biology on the Grid. *J. Grid Comput.* **2012**, *10*, 743–767.

2. Figures

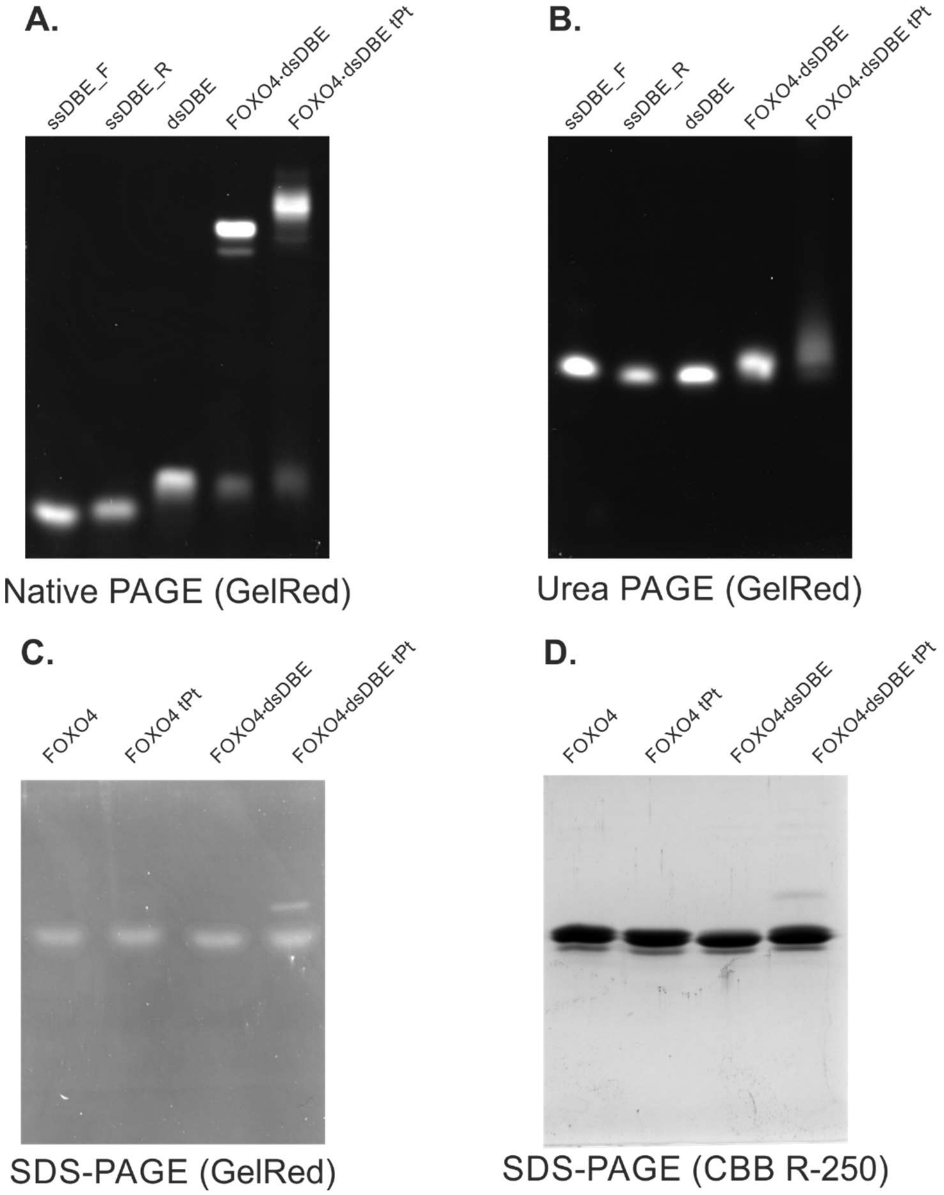

Figure S1: Native and denaturing polyacrylamide gel (12%) electrophoresis (PAGE) analysis of transplatinated FOXO4-DBD•DBE complex. (**A.**) Native analysis in TBE buffer. (**B.**) Denaturing analysis in TBE and urea buffer. (**C.**) and **D.**) Denaturing analysis in TG-SDS buffer. The gels were stained by GelRed for nucleic acids visualization (**A.**, **B.**, **C.**) or by Coomassie brilliant blue R-250 (**D.**) for protein visualization. The individual strands constituting the DBE duplex were also analysed to facilitate data interpretation. A direct comparison of panel A. and B. or C. and D. enable one to immediately recognize the effects of protein-DNA cross-linking on the dissociation (or lack thereof) of FOXO4-DBD•DBE complex. Denotation FOXO4 corresponds to the FOXO4-DBD.

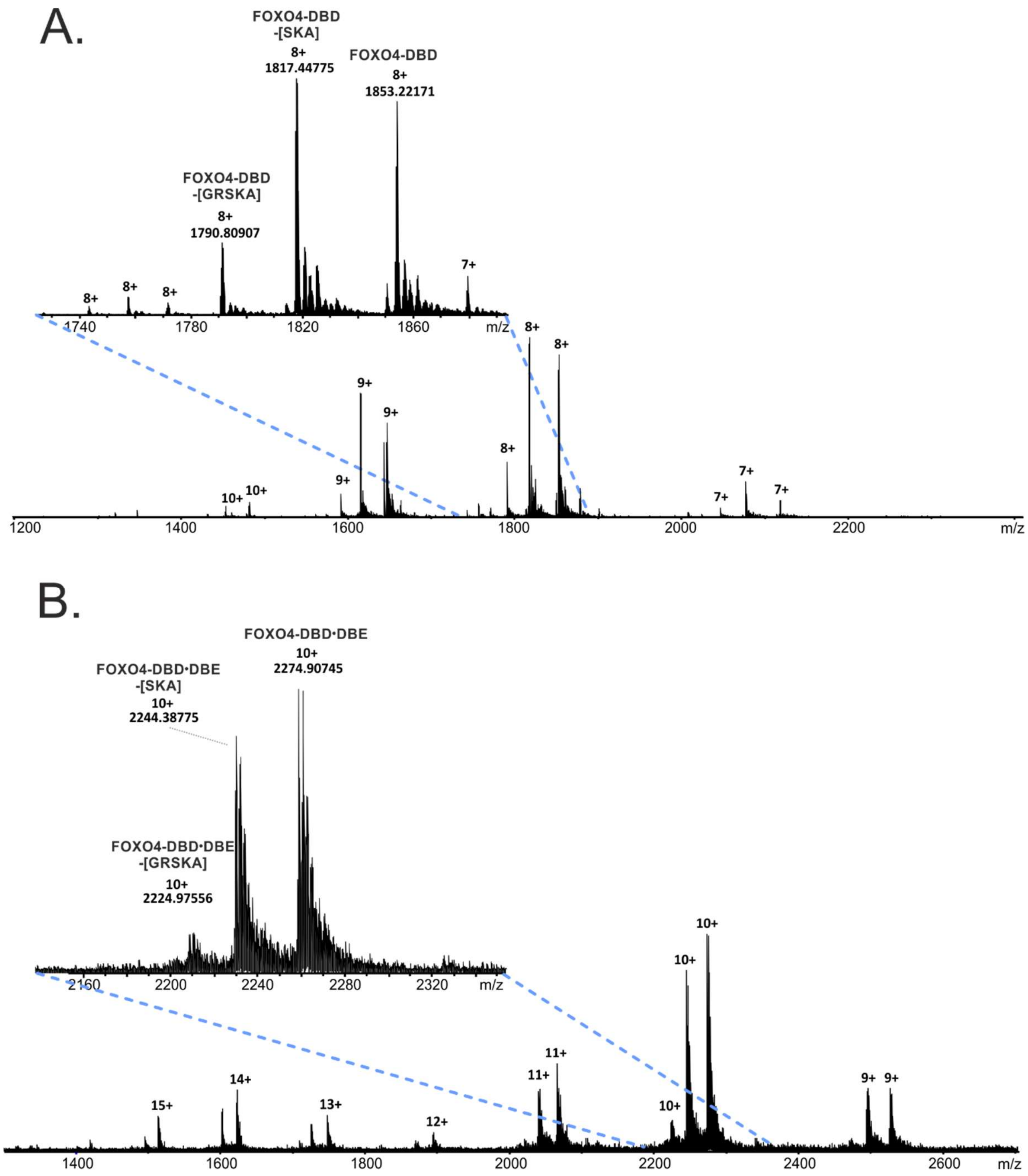

Figure S2: Native ESI-MS analysis of FOXO4-DBD•DBE complex. (**A.**) Mass spectrum of FOXO4-DBD and (**B.**) FOXO4-DBD•DBE obtained as described above. The initial recombinant material contained significant amounts of shorter versions of FOXO4-DBD produced by spontaneous cleavages near the C-terminus of the sequence. As shown here, the presence of these degradation products did not adversely affect data interpretation.

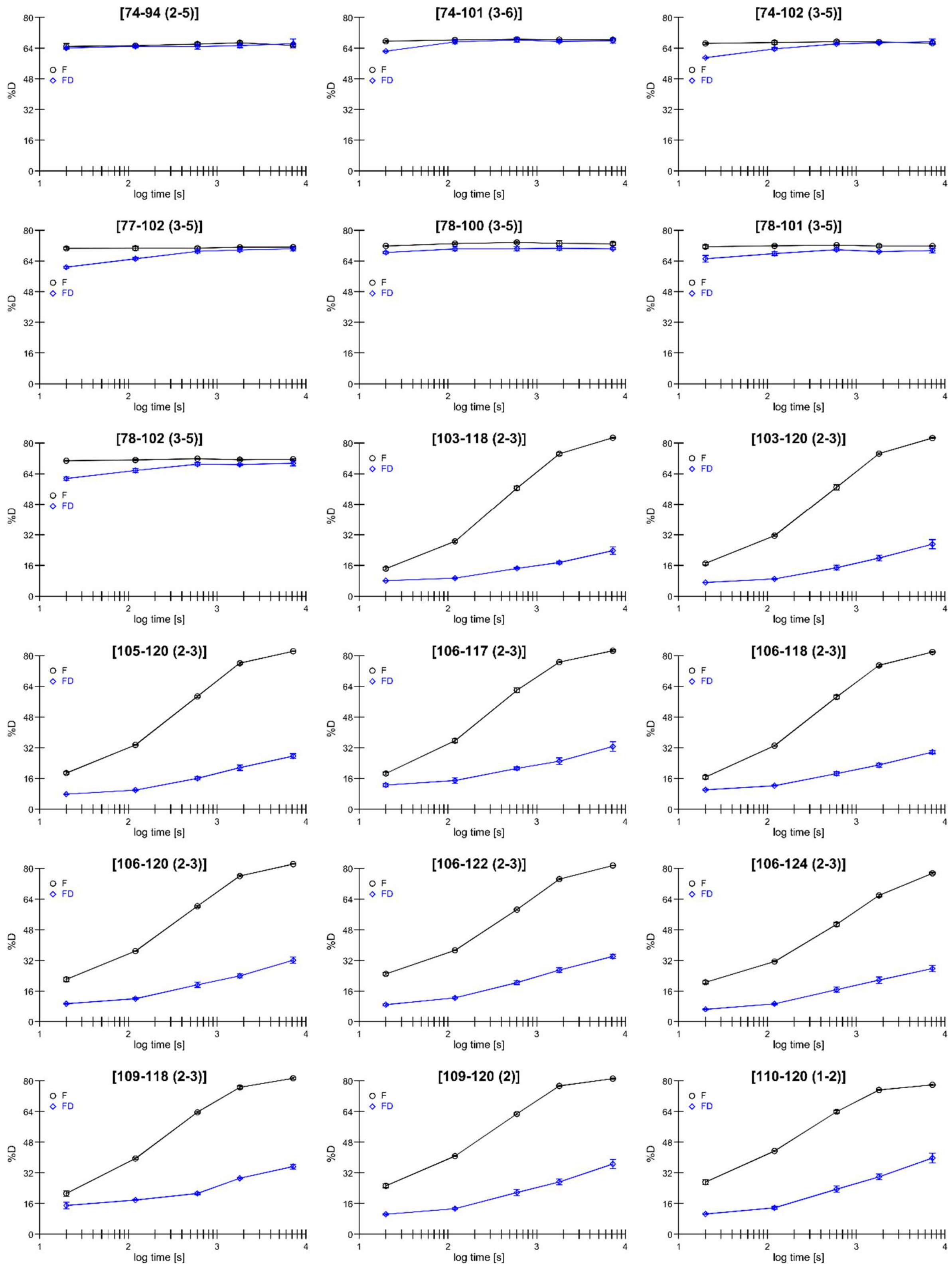

Figure S3: Relative deuteration rates of peptides obtained from either unbound FOXO4-DBD (F) or bound FOXO4-DBD•DBE (FD) complex (1/3). A direct comparison between corresponding data enables one to appreciate the effects of DBE binding on the solvent accessibility manifested by the various regions of the DBD. Numbers in square brackets correspond to the starting and ending amino acid positions in sequence. Number in round brackets represent the charge state(s) of peptide(s) used for calculation of relative deuteration.

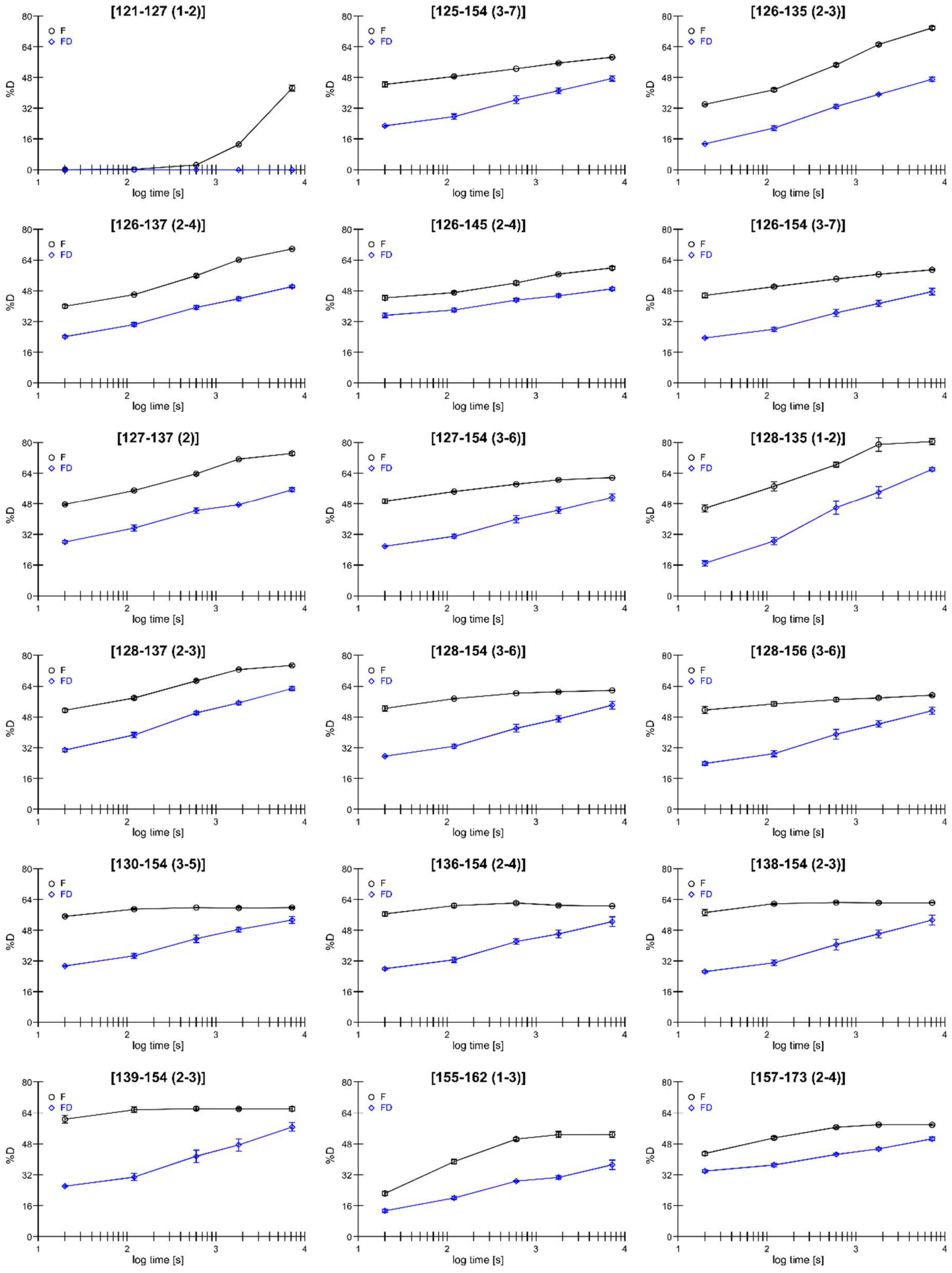

Figure S4: Relative deuteration rates of peptides obtained from either unbound FOXO4-DBD (F) or bound FOXO4-DBD•DBE (FD) complex (2/3). A direct comparison between corresponding data enables one to appreciate the effects of DBE binding on the solvent accessibility manifested by the various regions of the DBD. Number in round brackets represent the charge state(s) of peptide(s) used for calculation of relative deuteration.

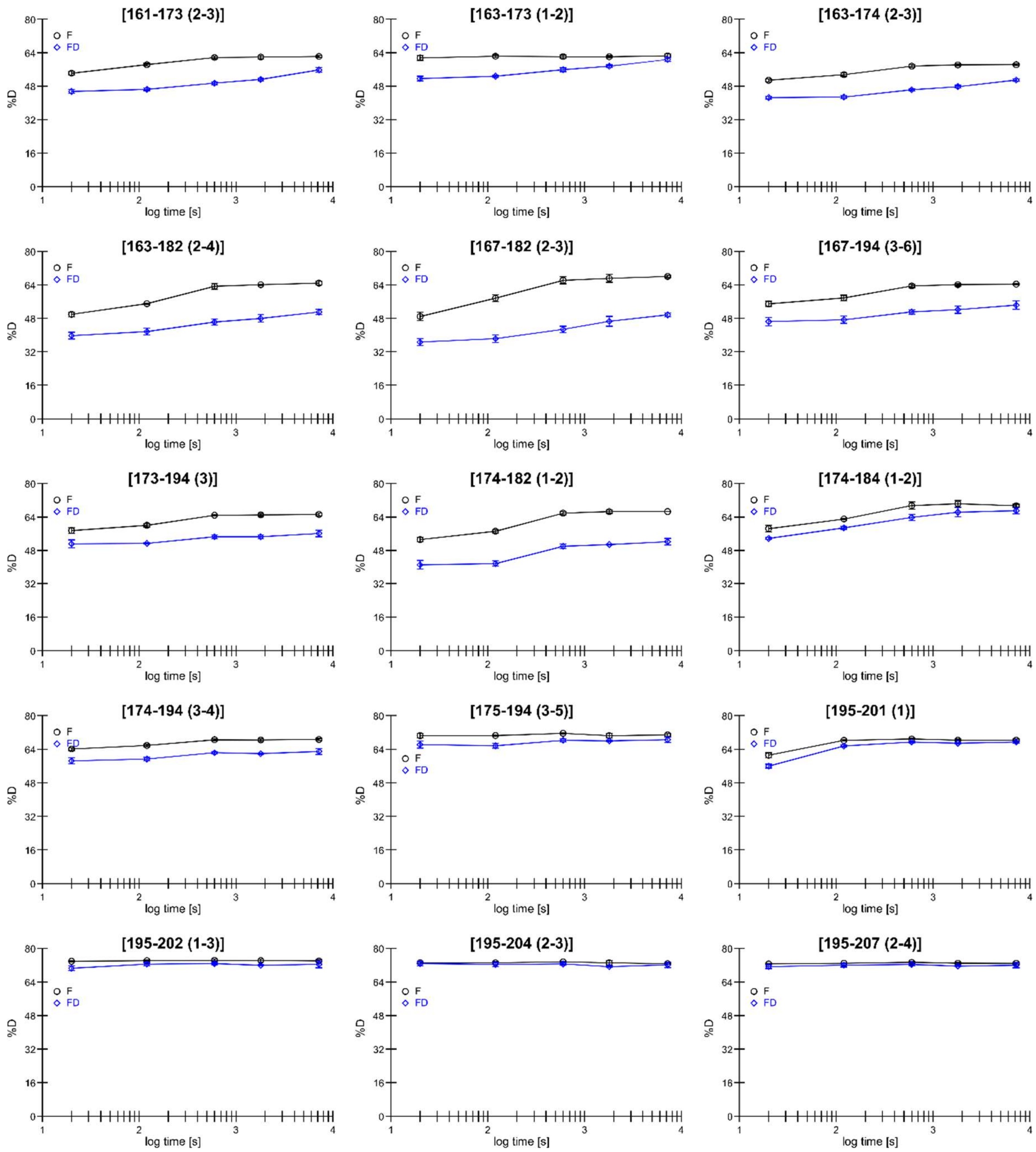

Figure S5: Relative deuteration rates of peptides obtained from either unbound FOXO4-DBD (F) or bound FOXO4-DBD•DBE (FD) complex (3/3). A direct comparison between corresponding data enables one to appreciate the effects of DBE binding on the solvent accessibility manifested by the various regions of the DBD. Number in round brackets represent the charge state(s) of peptide(s) used for calculation of relative deuteration.

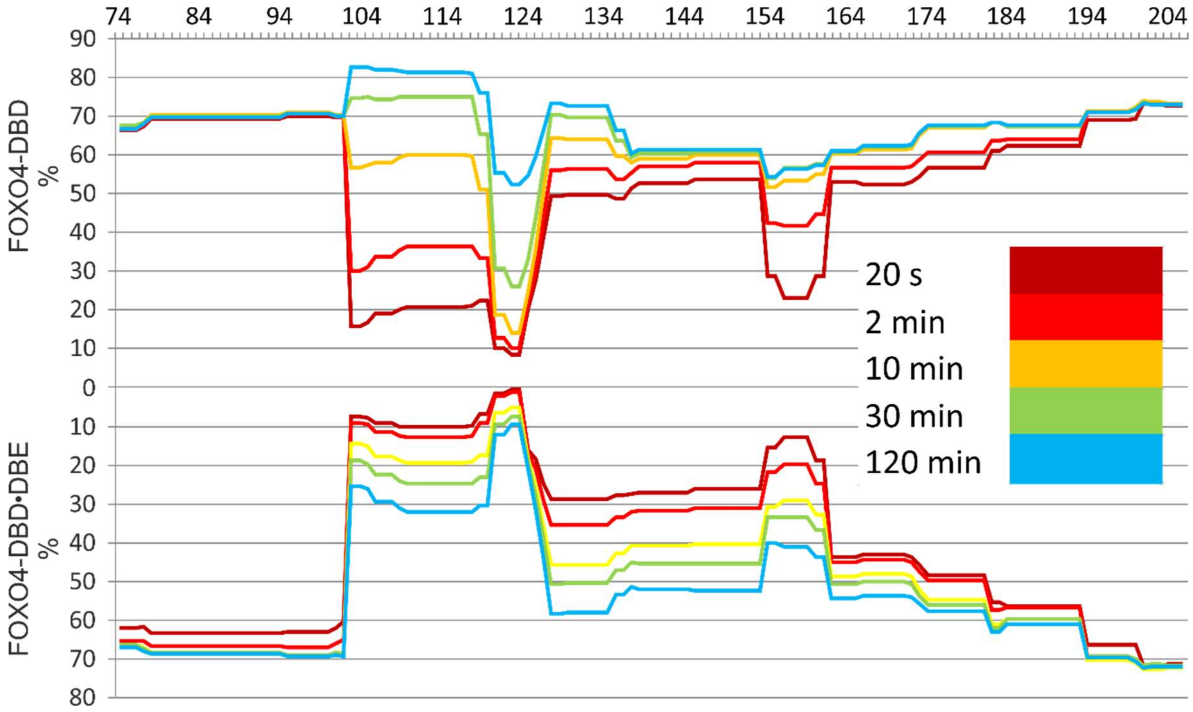

Figure S6: HDX butterfly plot. Time course of the relative deuteration rates of FOXO4-DBD and FOXO4-DBD•DBE complex plotted along the protein sequence. Note that the G_{74} - P_{87} region of our construct was introduced by the vector used for recombinant production, and thus was not part of the wildtype FOXO4 sequence (see **Supporting Information Figure S14**).

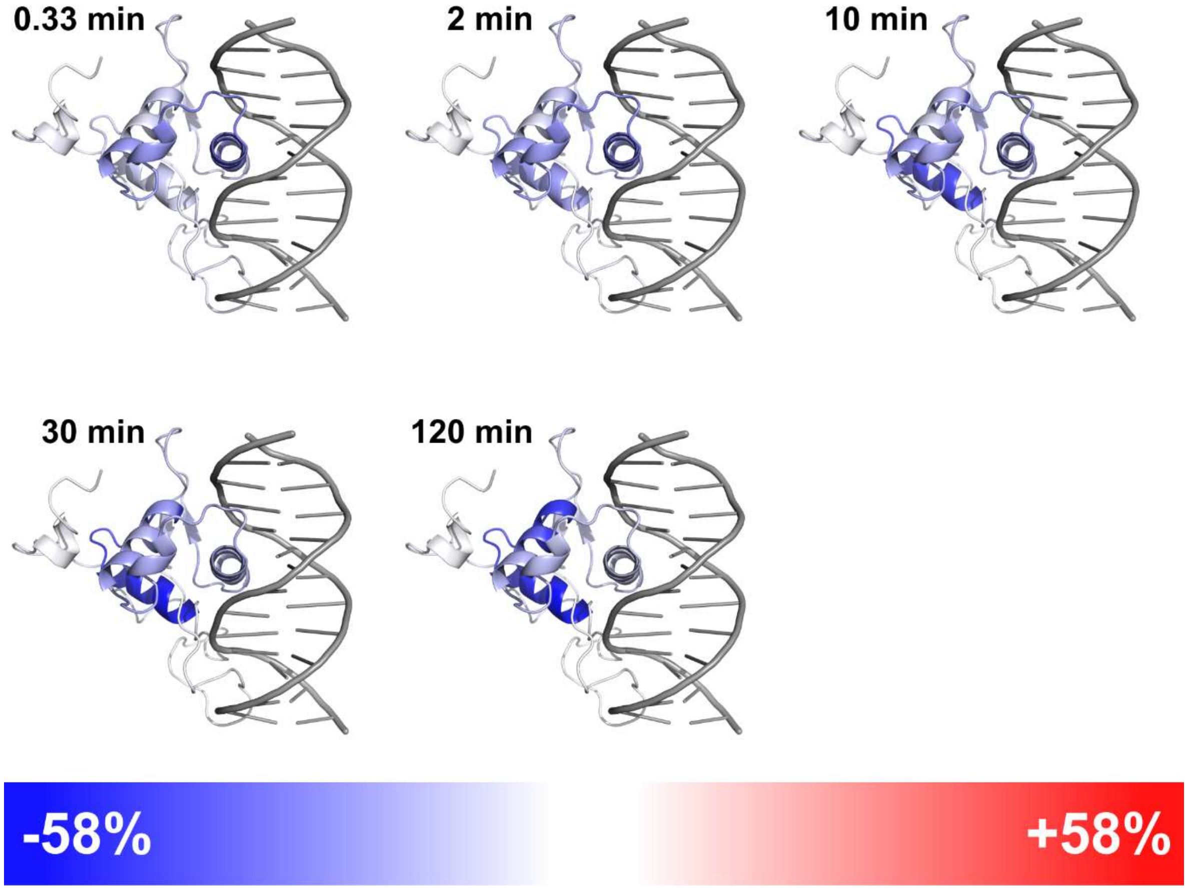

Figure S7: Variations of relative deuteration rates as a function of time mapped onto the protein structure. Blue-white-red spectrum bar represents the levels of deuteration difference between unbound and bound state (unbound – bound). Major variations were readily detectable not only in the regions involved in direct contacts with the DBE component, but also in regions that were distal to the binding site, thus suggesting possible long-distance allosteric effects.

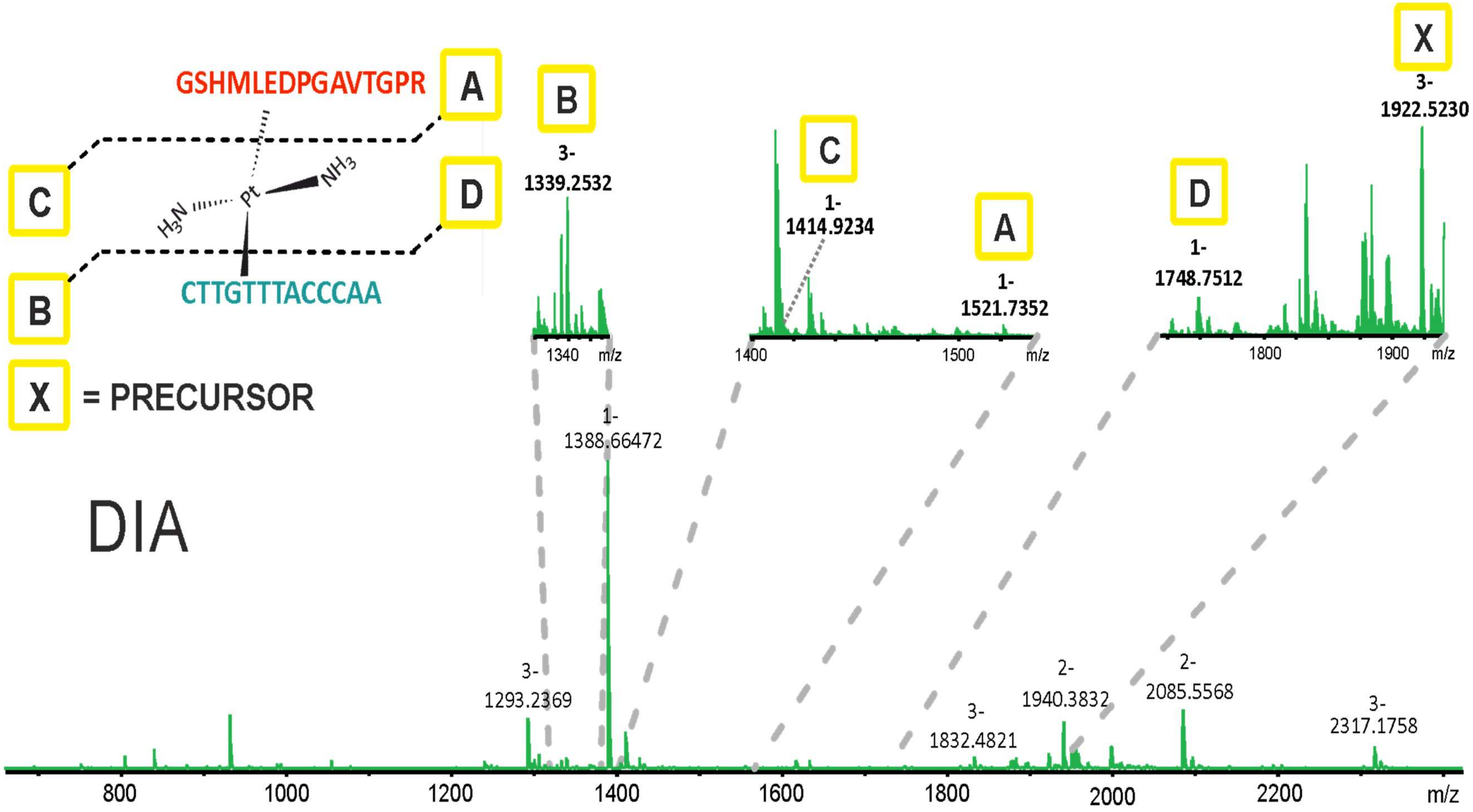

Figure S8: Collision-induced dissociation (CID) of peptide-oligonucleotide cross-link by transplatin. This is a representative spectrum illustrating the results of data-independent analysis performed in negative ion mode. Fragmentation was obtained by using 15 eV fixed CID energy. The scheme provides data interpretation showing the position of the transplatin bridge in this peptide-oligonucleotide conjugate.

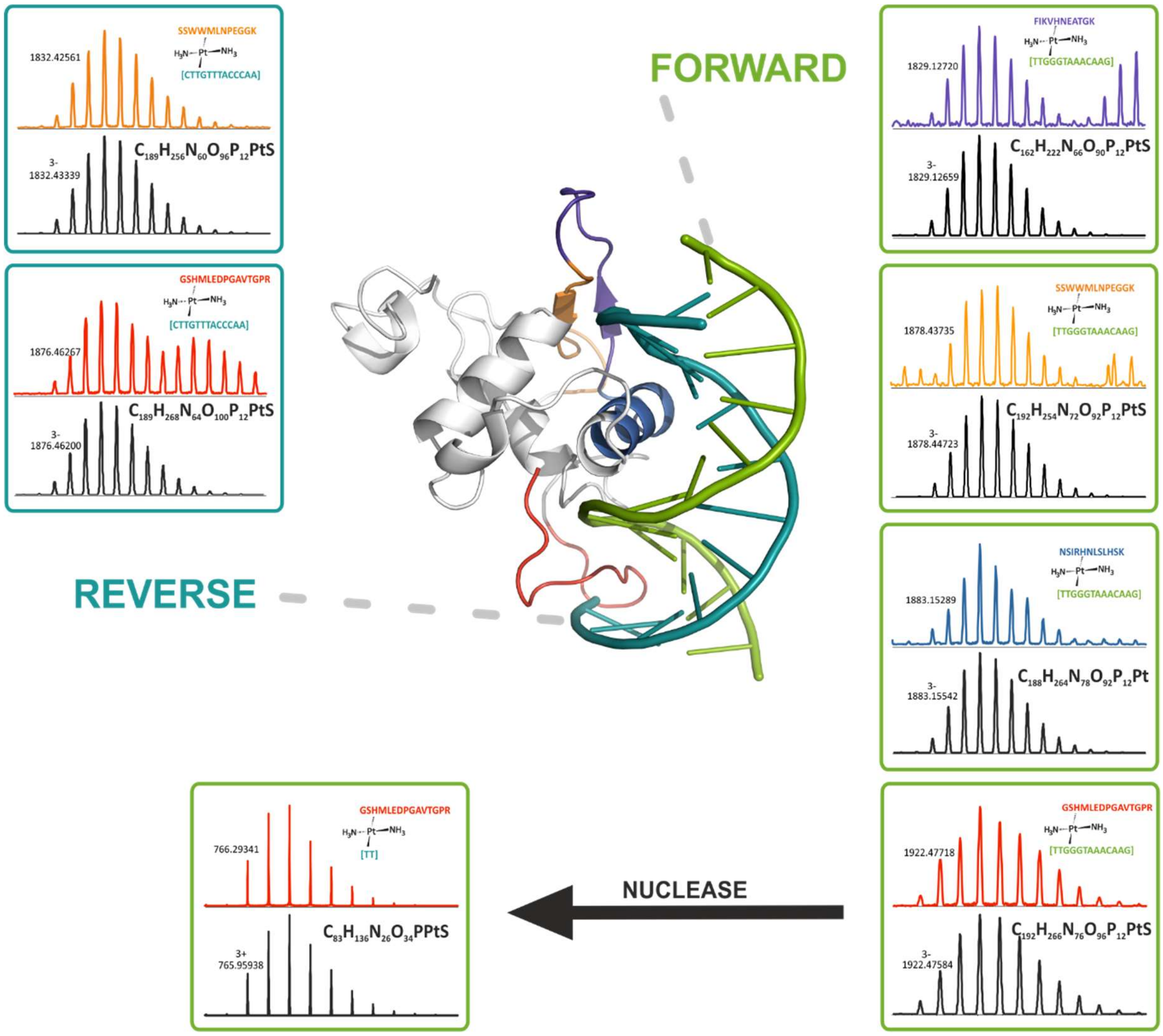

Figure S 9: Representative data obtained from protein-DNA cross-linking by transplatin. The boxes display different peptide-oligonucleotide species produced by trypsin digestion. The upper spectrum in each box represents experimental data that are color-coded with the corresponding region of the FOXO4-DBD•DBE homology model. The bottom part represents the theoretical isotopic distribution predicted for the corresponding conjugate as described above. The boxes are color-coded to match the corresponding contours of the nucleic acid strands in the homology model.

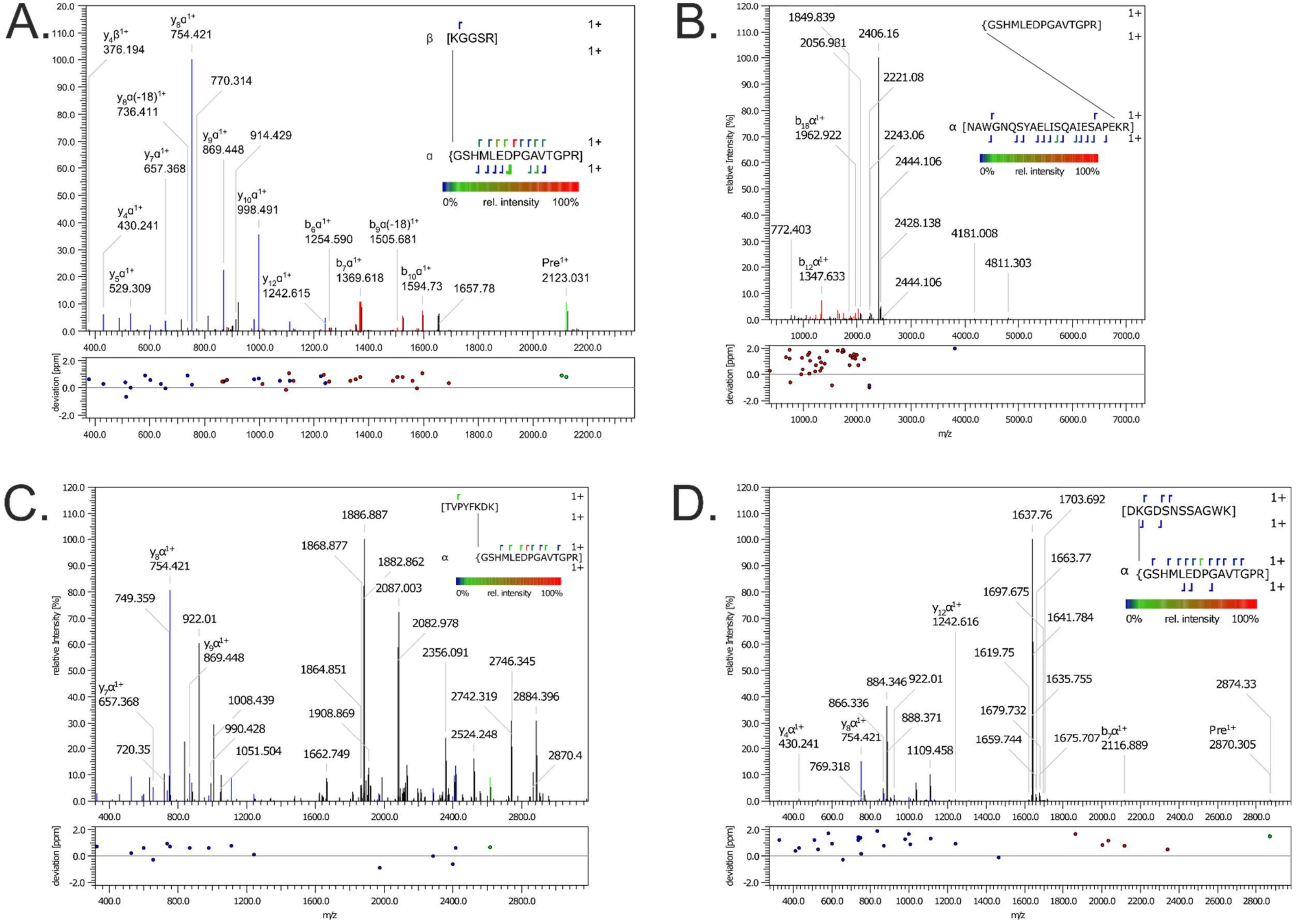

Figure S 10: Mass spectra of cross-links dissociation products (1/17). Each spectrum represents a single identified peptide-peptide cross-linked by DSG [di(N-succinimidyl) glutarate] (represented by line connecting two cross-linked residues): **A.** N-term. - K_{89}; **B.** N-term. - K_{116}; **C.** N-term. - K_{135}; **D.** N-term. - K_{137}. Fragmentation sites are marked along the amino acids sequence of each peptide. Resulting fragments are highlighted and designated in each spectrum.

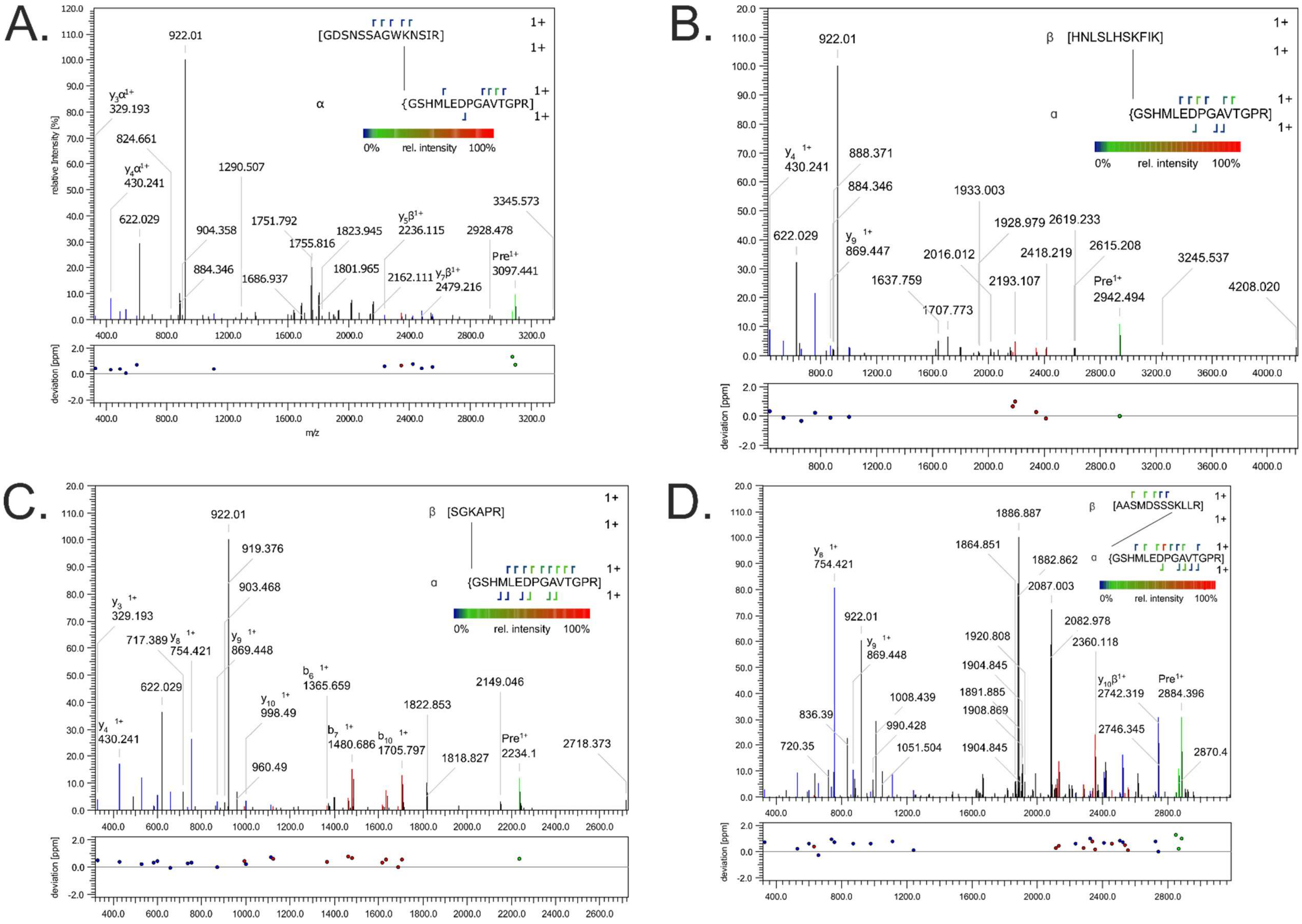

Figure S11: Mass spectra of cross-links dissociation products (2/17). Each spectrum represents a single identified peptide-peptide cross-linked by DSG [di(N-succinimidyl) glutarate] (represented by line connecting two cross-linked residues): **A.** N-term. - K_{147}; **B.** N-term. - K_{159}; **C.** N-term. - K_{185}; **D.** N-term. - K_{199}. Fragmentation sites are marked along the amino acids sequence of each peptide. Resulting fragments are highlighted and designated in each spectrum.

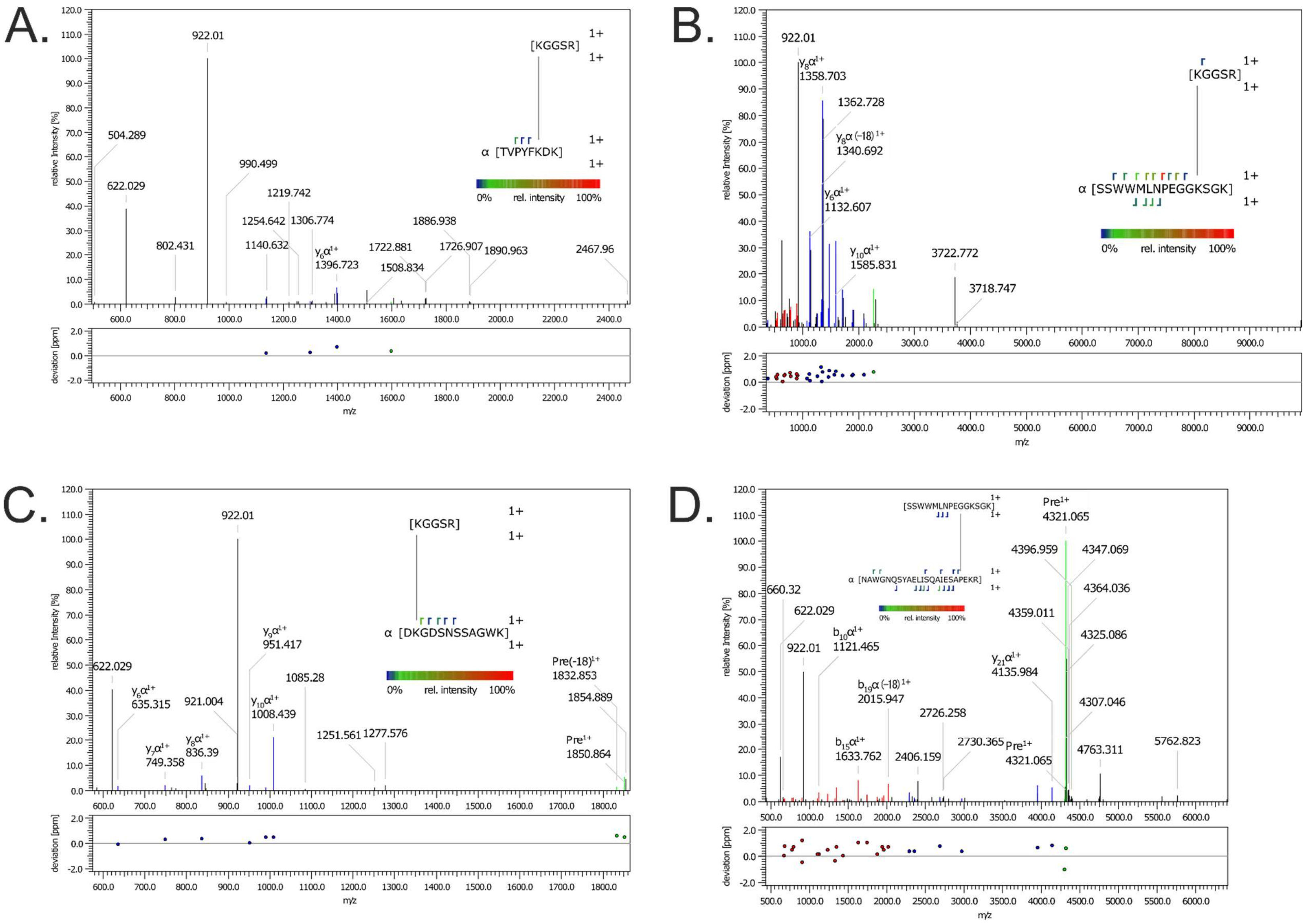

Figure S12: Mass spectra of cross-links dissociation products (3/17). Each spectrum represents a single identified peptide-peptide cross-linked by DSG [di(N-succinimidyl) glutarate] (represented by line connecting two cross-linked residues): **A.** K_{89} - K_{135}; **B.** K_{89} - K_{182}; **C.** K_{89} - K_{199}; **D.** K_{116} - K_{182}. Fragmentation sites are marked along the amino acids sequence of each peptide. Resulting fragments are highlighted and designated in each spectrum.

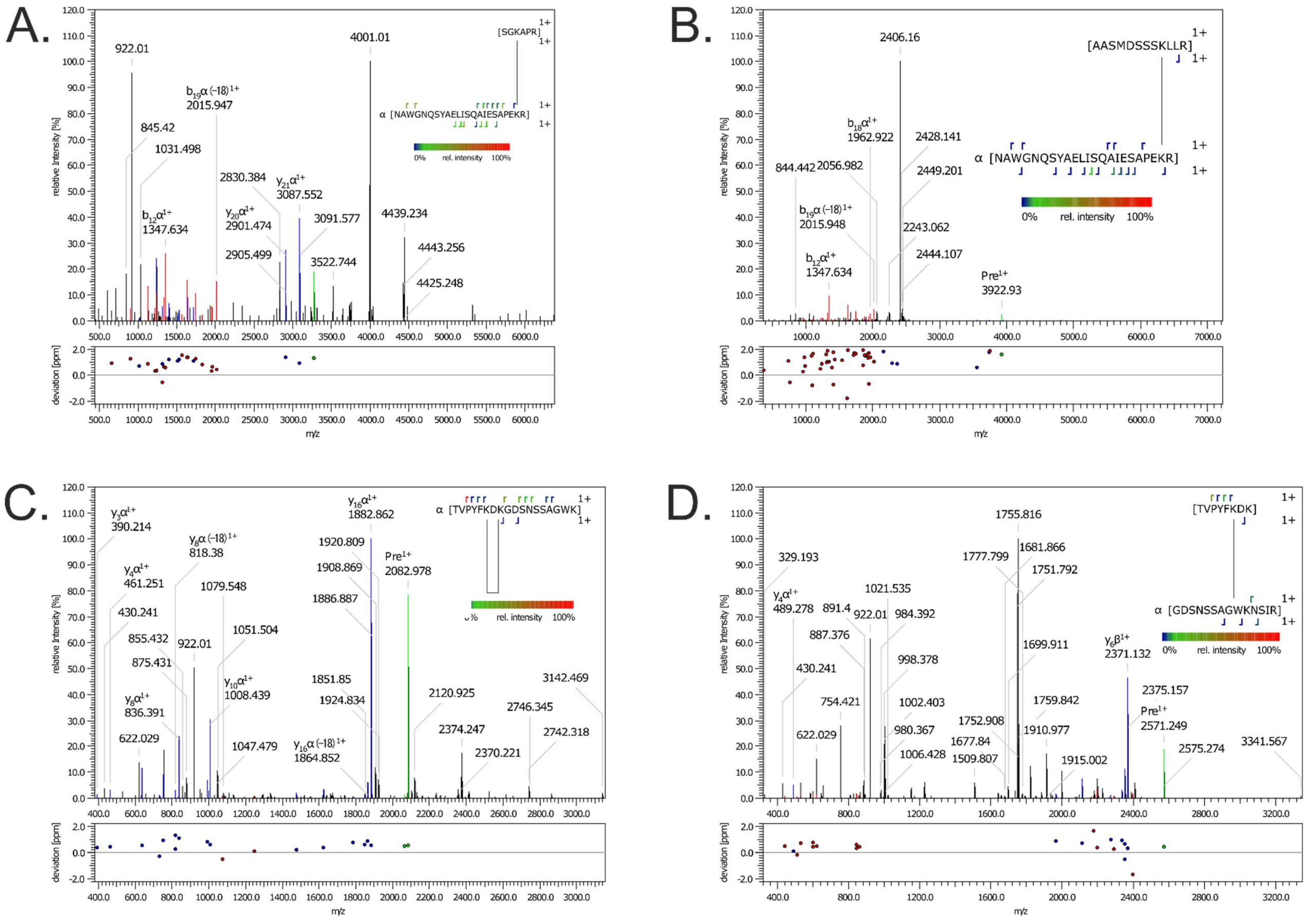

Figure S13: Mass spectra of cross-links dissociation products (4/17). Each spectrum represents a single identified peptide-peptide cross-linked by DSG [di(N-succinimidyl) glutarate] (represented by line connecting two cross-linked residues): **A.** K116 - K185; **B.** K116 - K199; **C.** K135 - K137; **D.** K135 - K147. Fragmentation sites are marked along the amino acids sequence of each peptide. Resulting fragments are highlighted and designated in each spectrum.

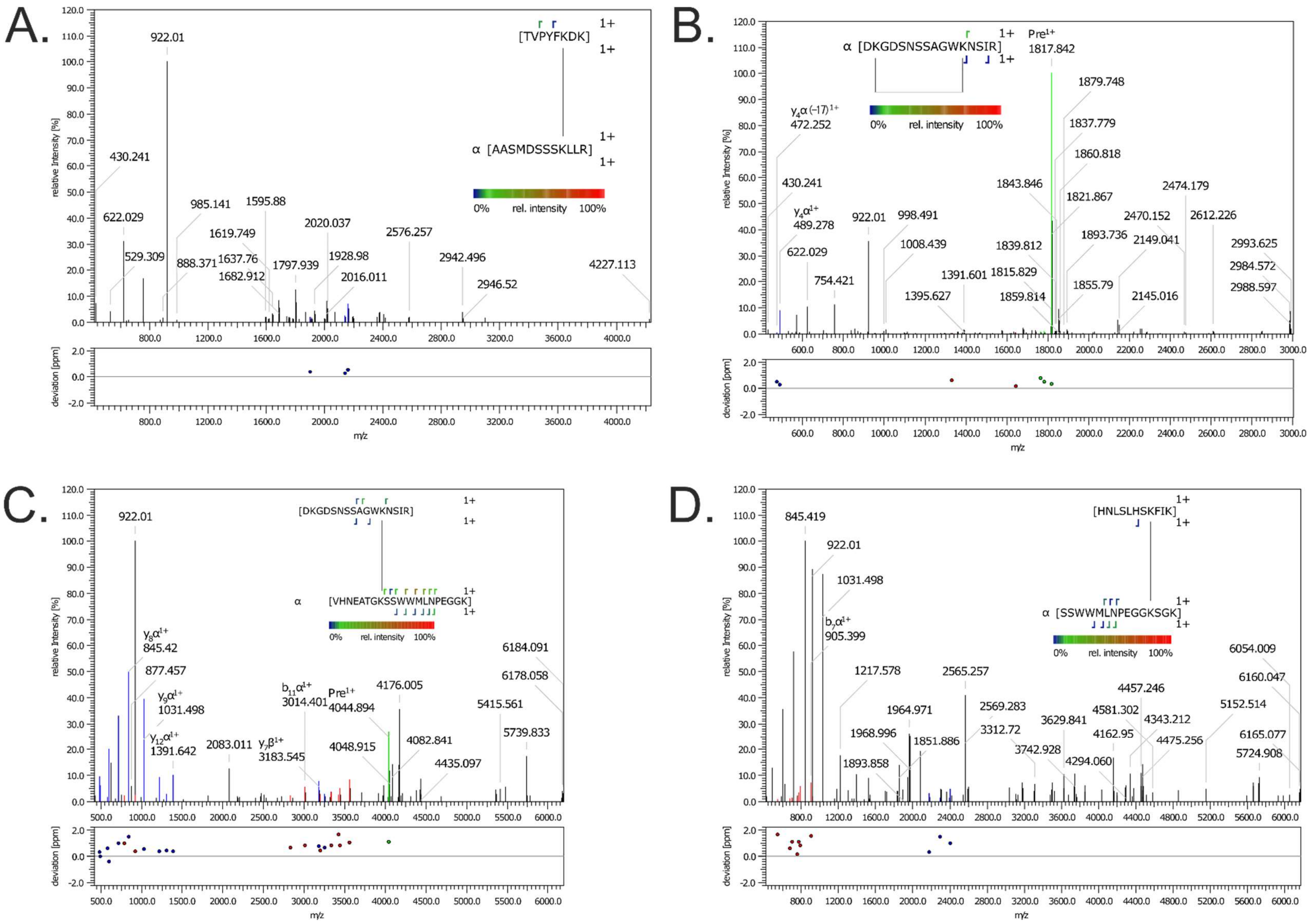

Figure S14: Mass spectra of cross-links dissociation products (5/17). Each spectrum represents a single identified peptide-peptide cross-linked by DSG [di(N-succinimidyl) glutarate] (represented by line connecting two cross-linked residues): **A.** K_{135} - K_{199}; **B.** K_{137} - K_{147}; **C.** K_{147} - K_{170}; **D.** K_{159} - K_{182}. Fragmentation sites are marked along the amino acids sequence of each peptide. Resulting fragments are highlighted and designated in each spectrum.

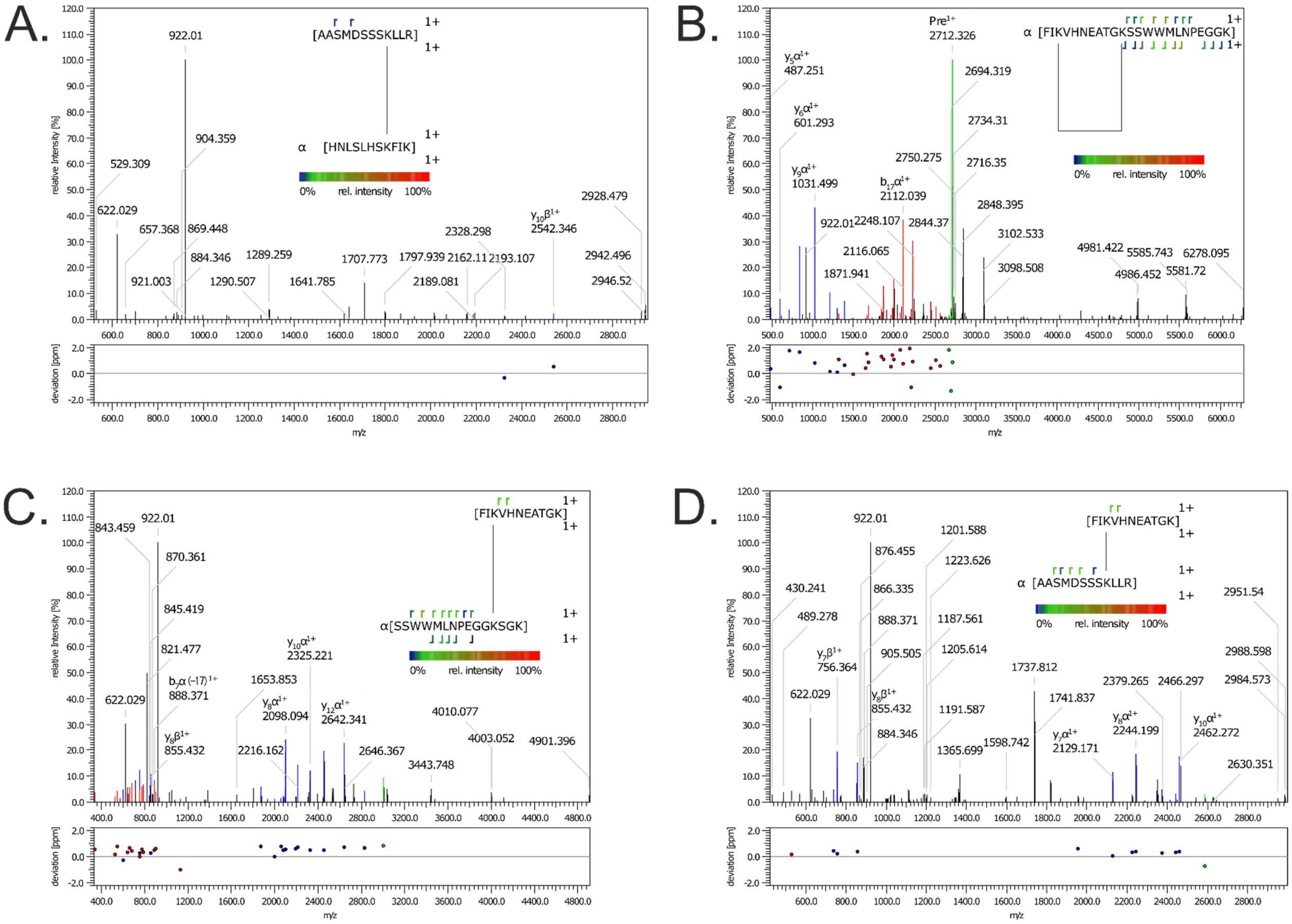

Figure S15: Mass spectra of cross-links dissociation products (6/17). Each spectrum represents a single identified peptide-peptide cross-linked by DSG [di(N-succinimidyl) glutarate] (represented by line connecting two cross-linked residues): **A.** K_{159} - K_{199}; **B.** K_{162}-K_{170}; **C.** K_{162}-K_{182}; **D.** K_{162}-K_{199}. Fragmentation sites are marked along the amino acids sequence of each peptide. Resulting fragments are highlighted and designated in each spectrum.

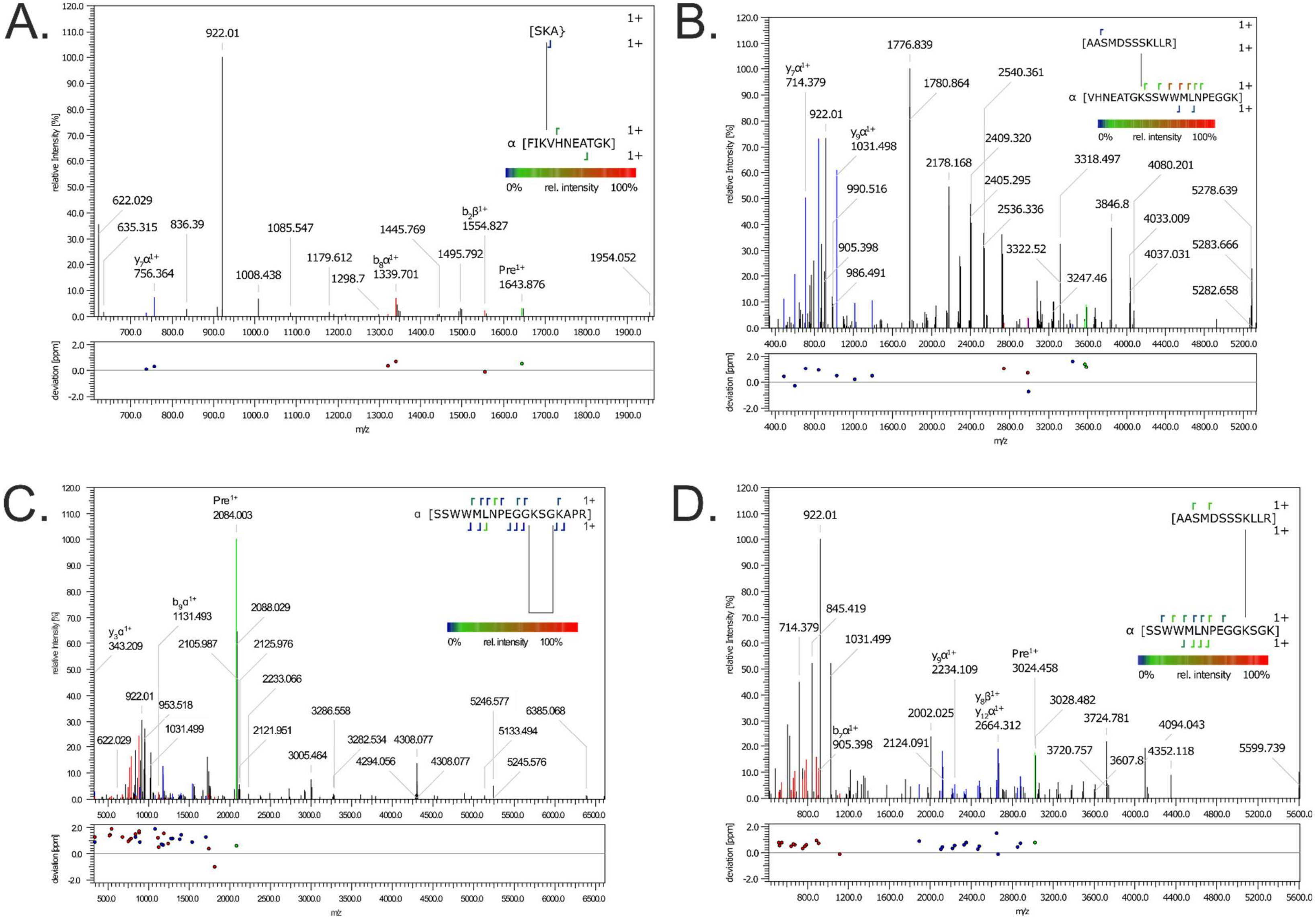

Figure S16: Mass spectra of cross-links dissociation products (7/17). Each spectrum represents a single identified peptide-peptide cross-linked by DSG [di(N-succinimidyl) glutarate] (represented by line connecting two cross-linked residues): **A.** K_{162} - K_{206}; **B.** K_{170} - K_{199}; **C.** K_{182} - K_{185}; **D.** K_{182} - K_{199}. Fragmentation sites are marked along the amino acids sequence of each peptide. Resulting fragments are highlighted and designated in each spectrum.

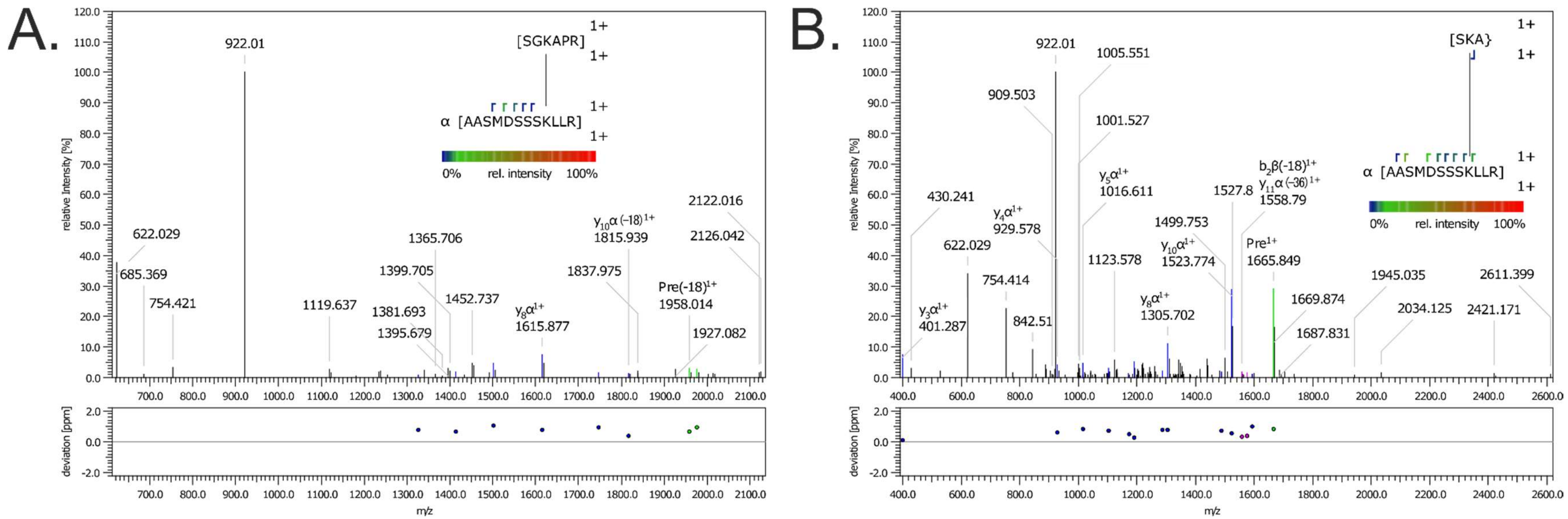

Figure S17: Mass spectra of cross-links dissociation products (8/17). Each spectrum represents a single identified peptide-peptide cross-linked by DSG [di(N-succinimidyl) glutarate] (represented by line connecting two cross-linked residues): **A.** K_{185} - K_{199}; **B.** K_{199} - K_{206}. Fragmentation sites are marked along the amino acids sequence of each peptide. Resulting fragments are highlighted and designated in each spectrum.

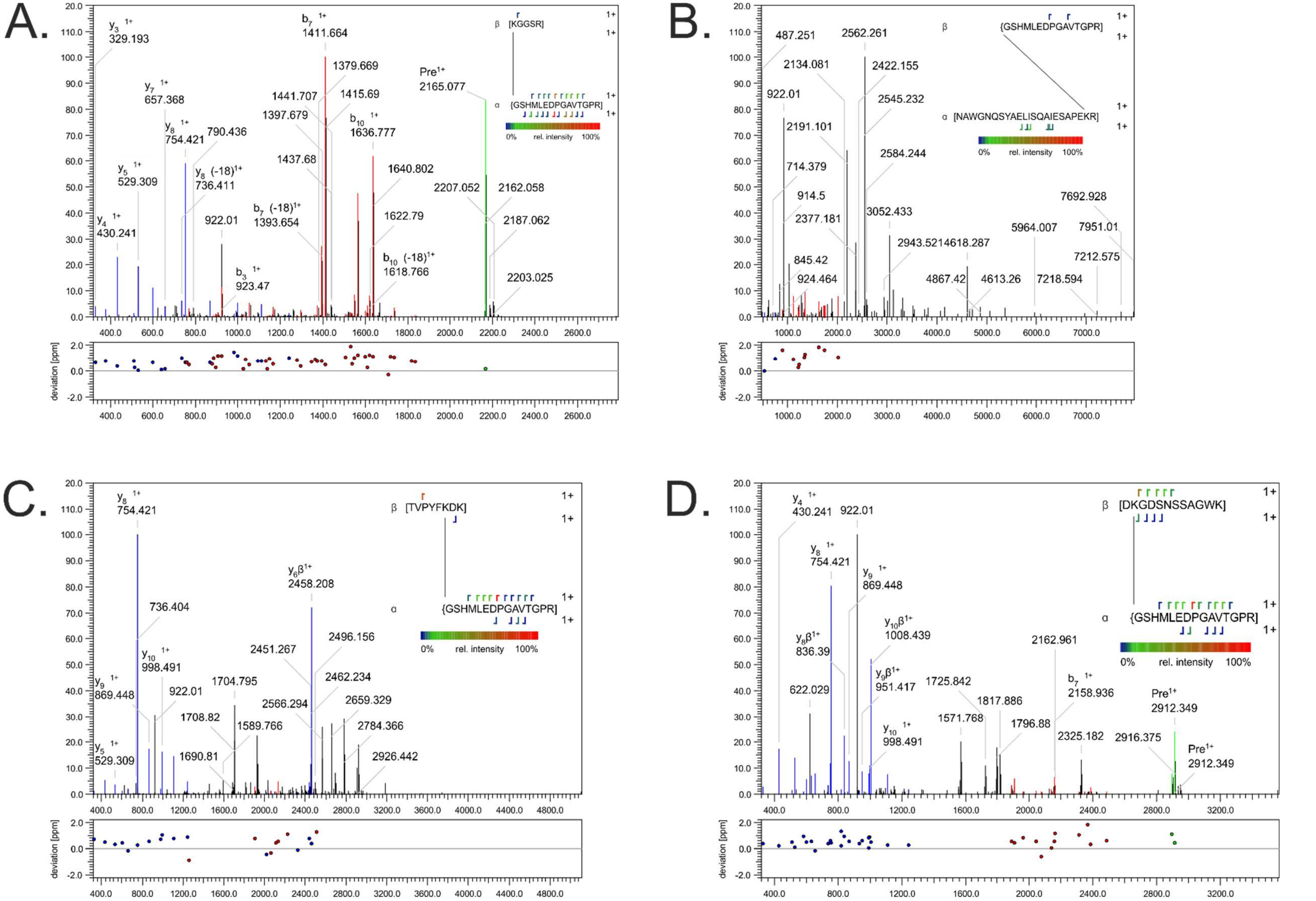

Figure S18: Mass spectra of cross-links dissociation products (9/17). Each spectrum represents a single identified peptide-peptide cross-linked by DSS [di(N-succinimidyl) suberate] (represented by line connecting two cross-linked residues): **A.** N-term. - K_{89}; **B.** N-term. - K_{116}; **C.** N-term. - K_{135}; **D.** N-term. - K_{137}. Fragmentation sites are marked along the amino acids sequence of each peptide. Resulting fragments are highlighted and designated in each spectrum.

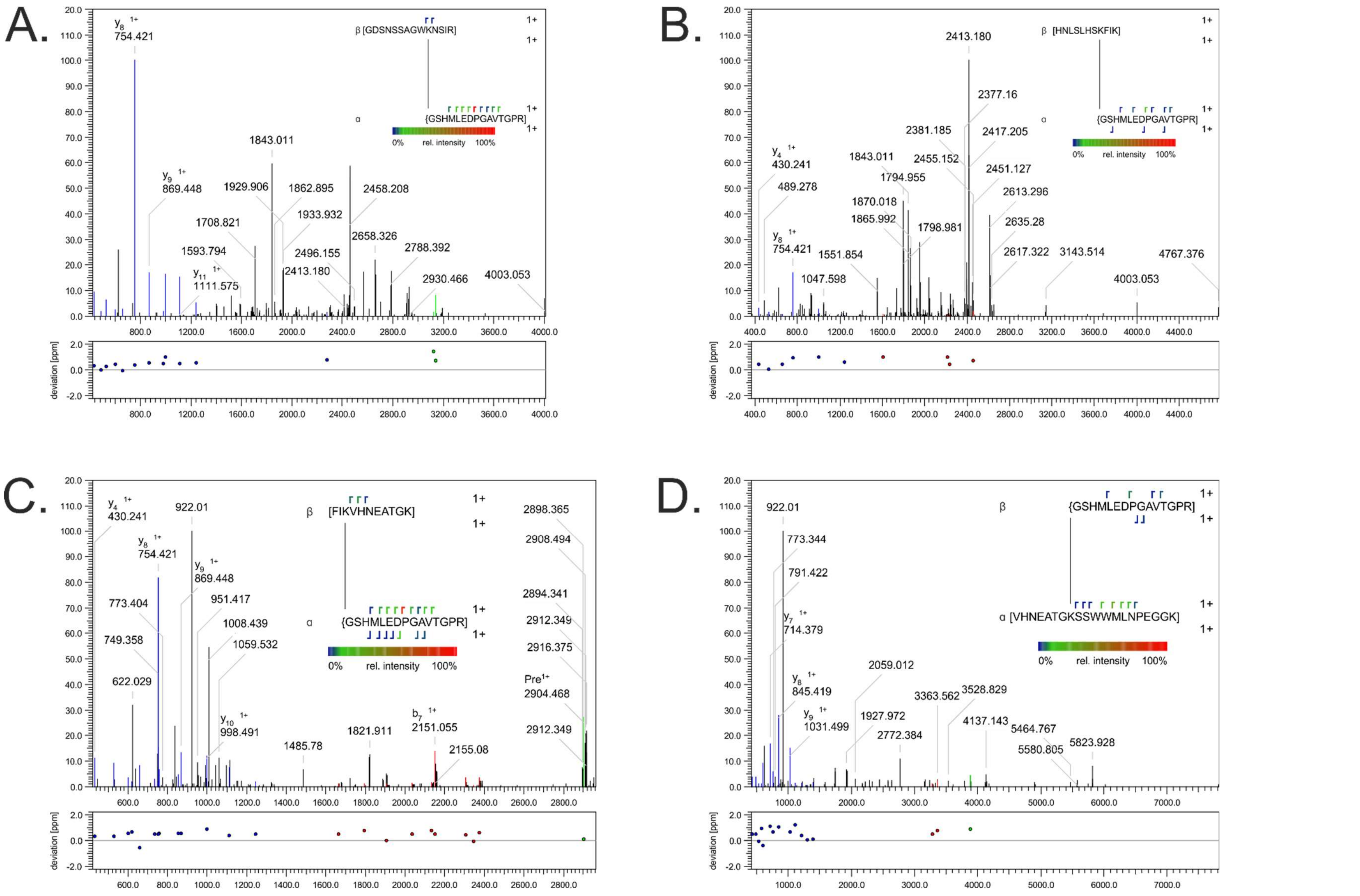

Figure S19: Mass spectra of cross-links dissociation products (10/17). Each spectrum represents a single identified peptide-peptide cross-linked by DSS [di(N-succinimidyl) suberate] (represented by line connecting two cross-linked residues): **A.** N-term. - K147; **B.** N-term. - K159; **C.** N-term. - K162; **D.** N-term. - K170. Fragmentation sites are marked along the amino acids sequence of each peptide. Resulting fragments are highlighted and designated in each spectrum.

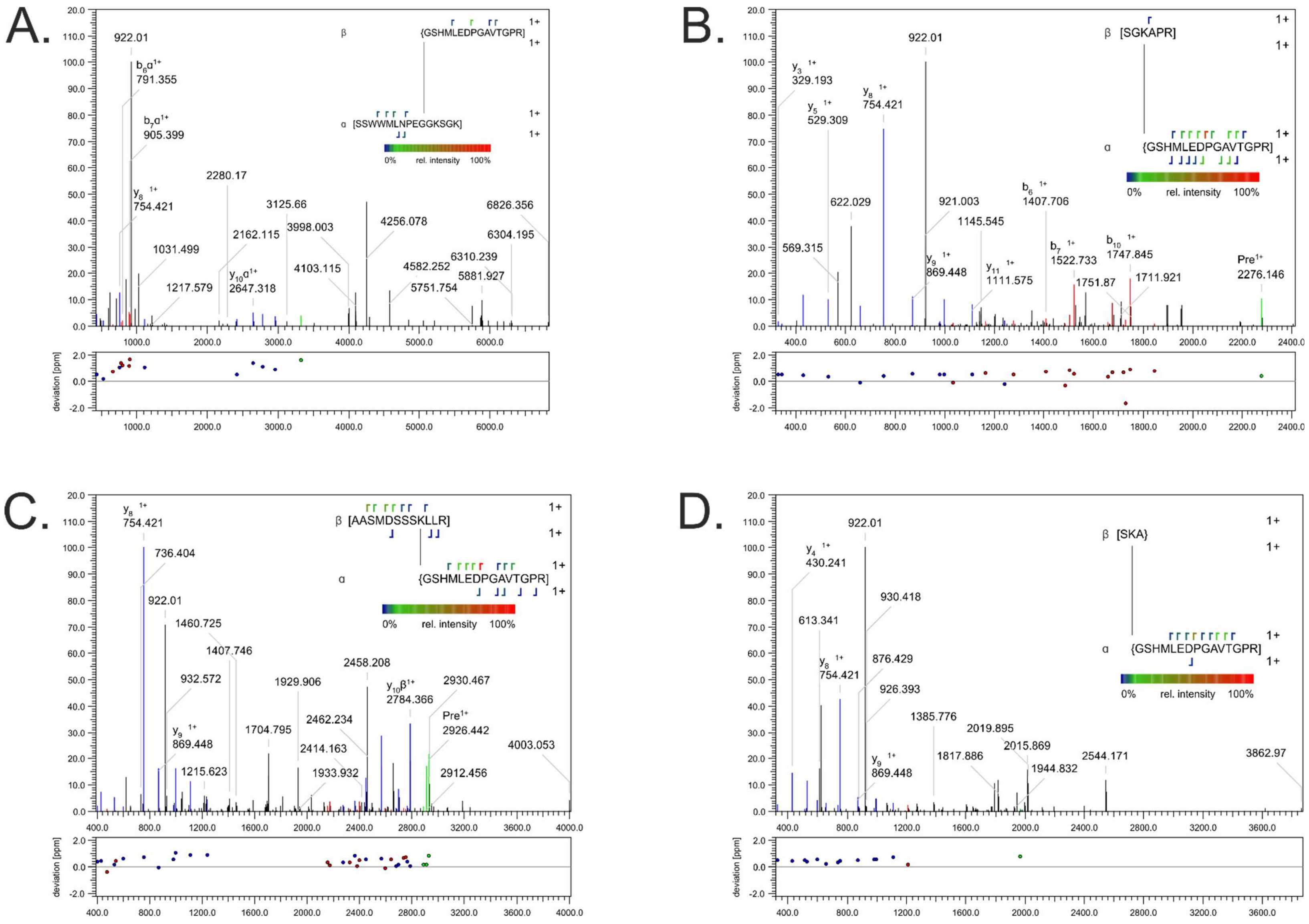

Figure S20: Mass spectra of cross-links dissociation products (11/17). Each spectrum represents a single identified peptide-peptide cross-linked by DSS [di(N-succinimidyl) suberate] (represented by line connecting two cross-linked residues): **A.** N-term. - K_{182}; **B.** N-term. - K_{185}; **C.** N-term. - K_{199}; **D.** N-term. - K_{206}. Fragmentation sites are marked along the amino acids sequence of each peptide. Resulting fragments are highlighted and designated in each spectrum.

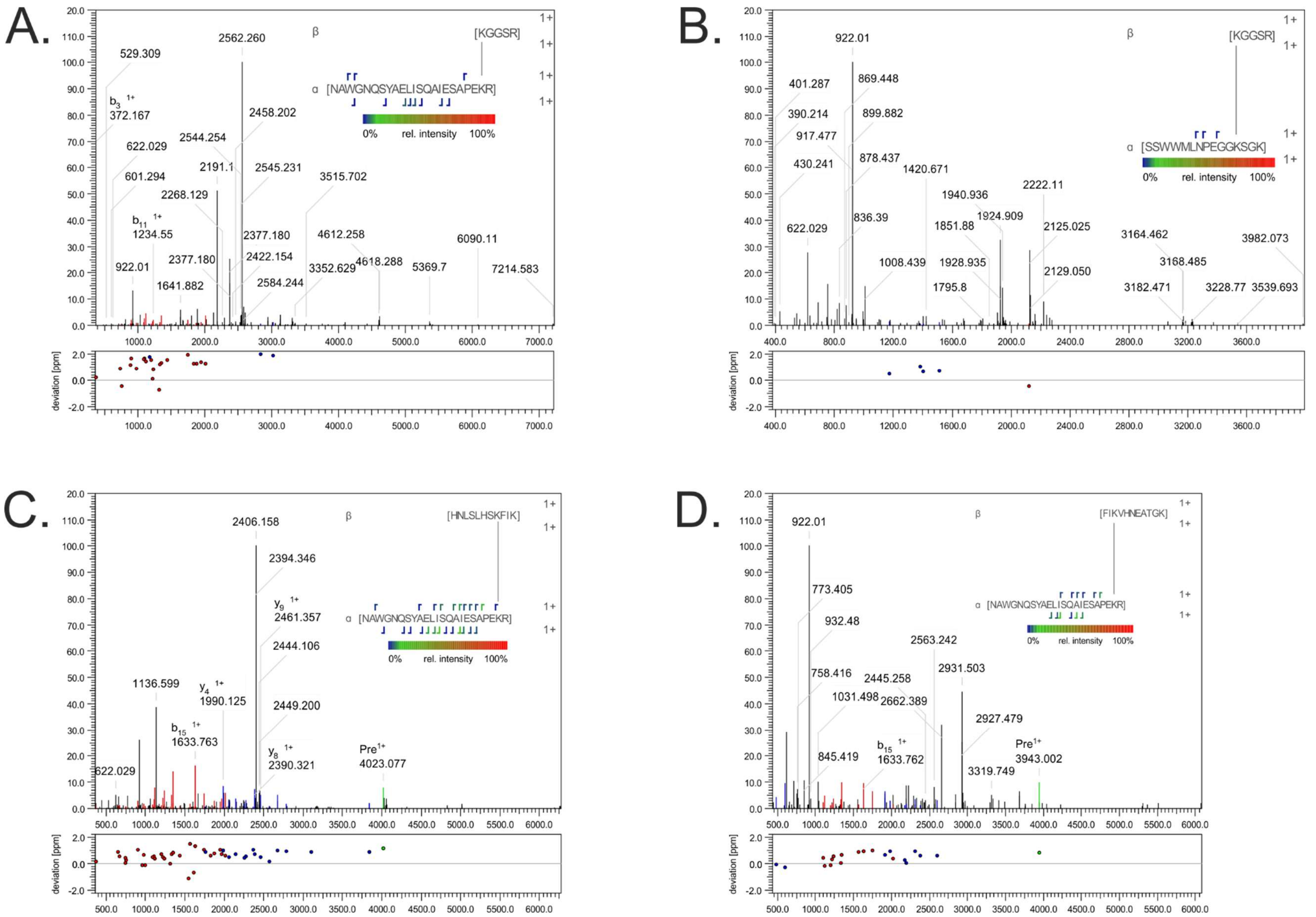

Figure S21: Mass spectra of cross-links dissociation products (12/17). Each spectrum represents a single identified peptide-peptide cross-linked by DSS [di(N-succinimidyl) suberate] (represented by line connecting two cross-linked residues): **A.** K_{89} - K_{116}; **B.** K_{89} - K_{182}; **C.** K_{116} - K_{159}; **D.** K_{116}-K_{162}. Fragmentation sites are marked along the amino acids sequence of each peptide. Resulting fragments are highlighted and designated in each spectrum.

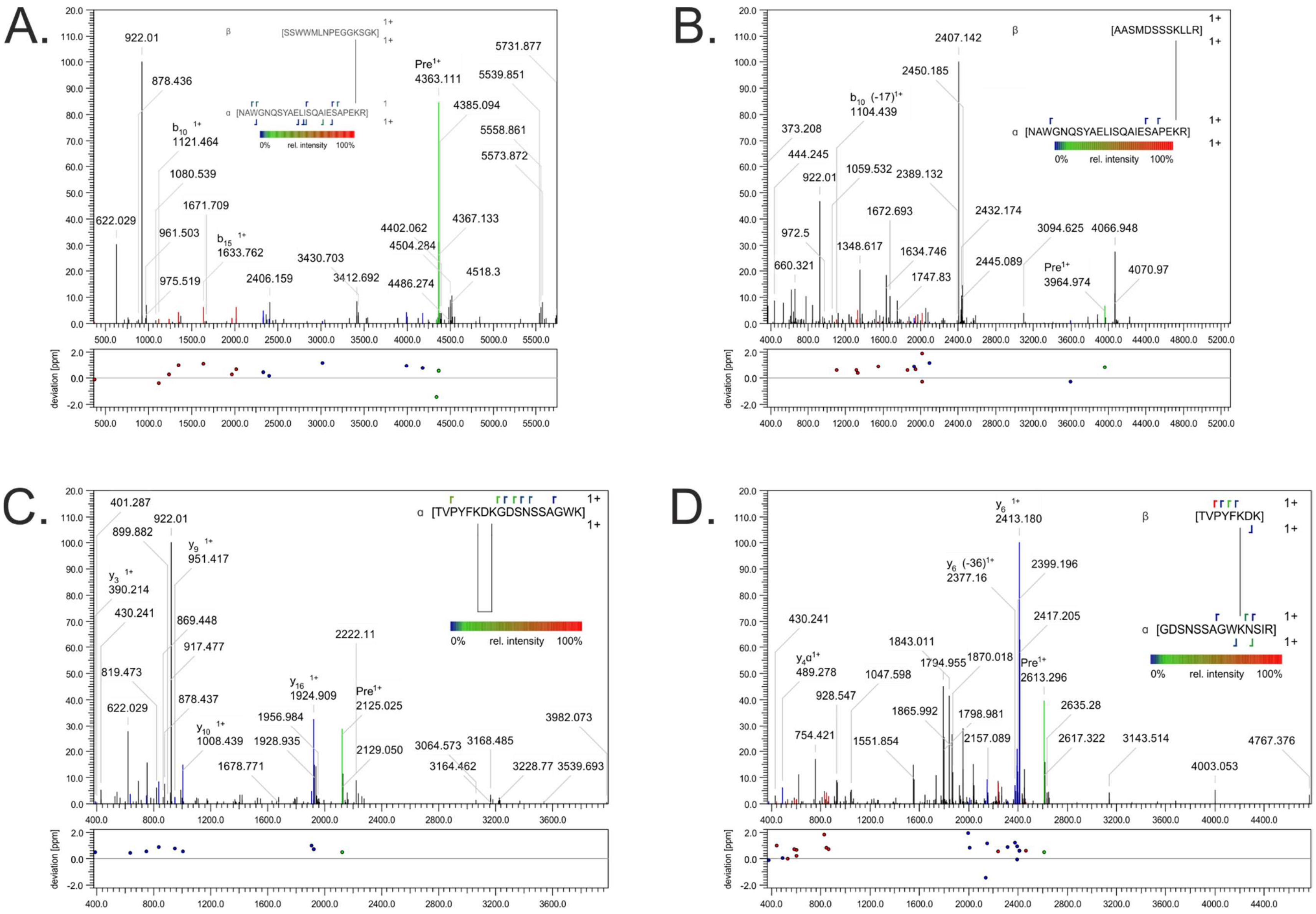

Figure S22: Mass spectra of cross-links dissociation products (13/17). Each spectrum represents a single identified peptide-peptide cross-linked by DSS [di(N-succinimidyl) suberate] (represented by line connecting two cross-linked residues): **A.** K_{116}-K_{182}; **B.** K_{116}-K_{199}; **C.** K_{135}-K_{137}; **D.** K_{135} - K_{147}. Fragmentation sites are marked along the amino acids sequence of each peptide. Resulting fragments are highlighted and designated in each spectrum.

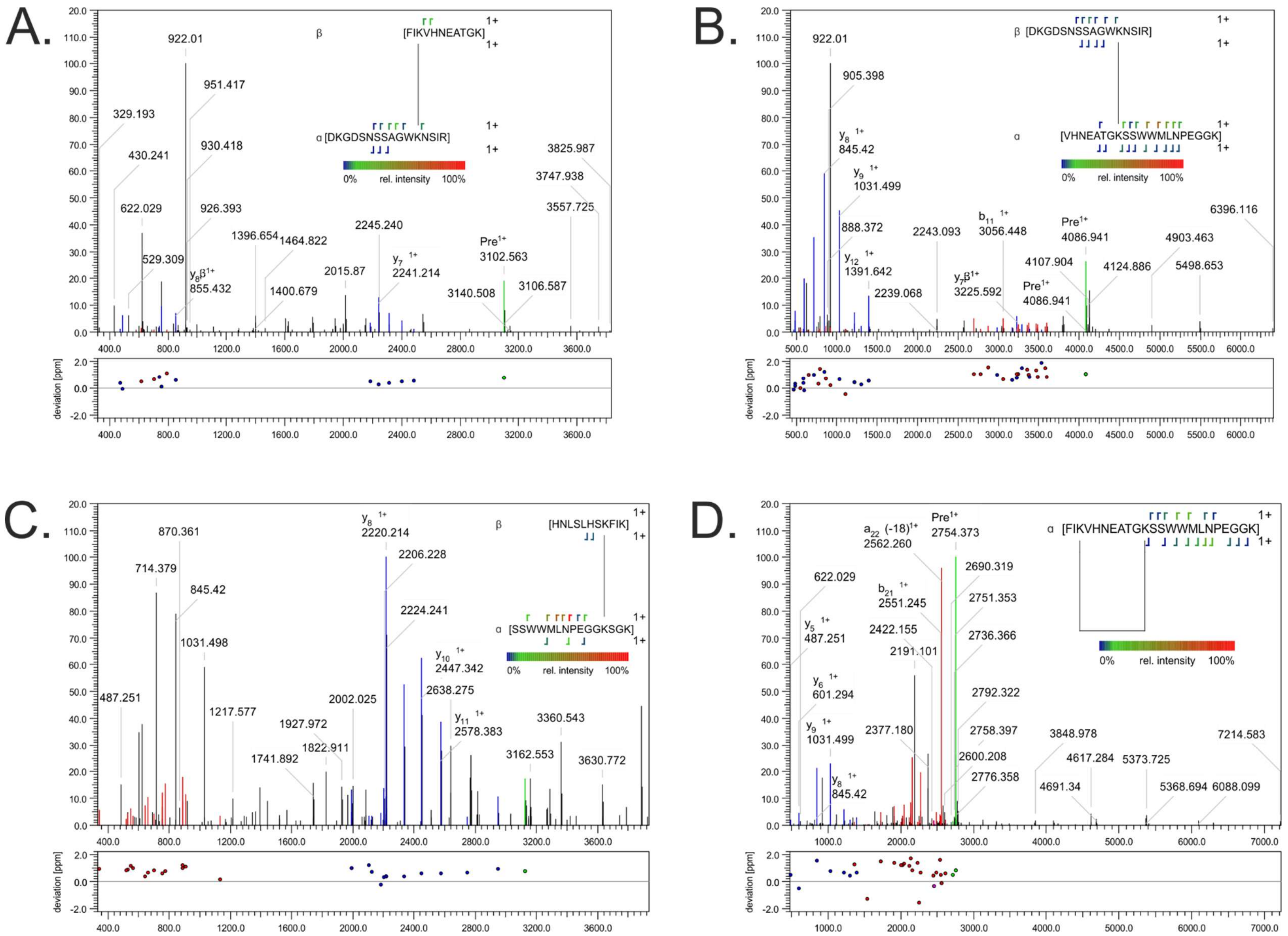

Figure S23: Mass spectra of cross-links dissociation products (14/17). Each spectrum represents a single identified peptide-peptide cross-linked by DSS [di(N-succinimidyl) suberate] (represented by line connecting two cross-linked residues): **A.** K_{147} - K_{162}; **B.** K_{147} - K_{170}; **C.** K_{159} - K_{182}; **D.** K_{162} - K_{170}. Fragmentation sites are marked along the amino acids sequence of each peptide. Resulting fragments are highlighted and designated in each spectrum.

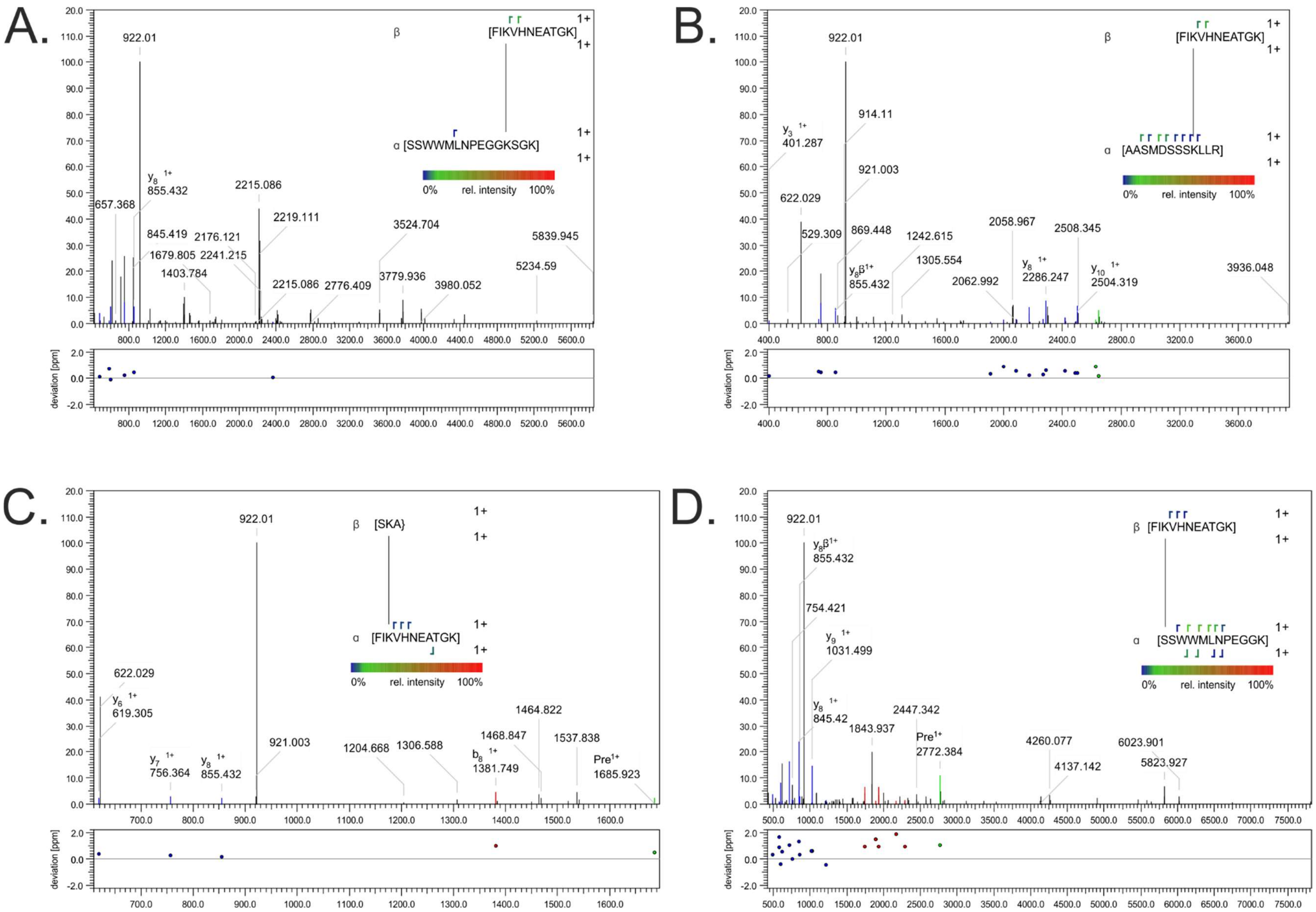

Figure S24: Mass spectra of cross-links dissociation products (15/17). Each spectrum represents a single identified peptide-peptide cross-linked by DSS [di(N-succinimidyl) suberate] (represented by line connecting two cross-linked residues): **A.** K_{162} - K_{182}; **B.** K_{162} - K_{199}; **C.** K_{162} - K_{206}; **D.** K_{162}-S_{172}. Fragmentation sites are marked along the amino acids sequence of each peptide. Resulting fragments are highlighted and designated in each spectrum.

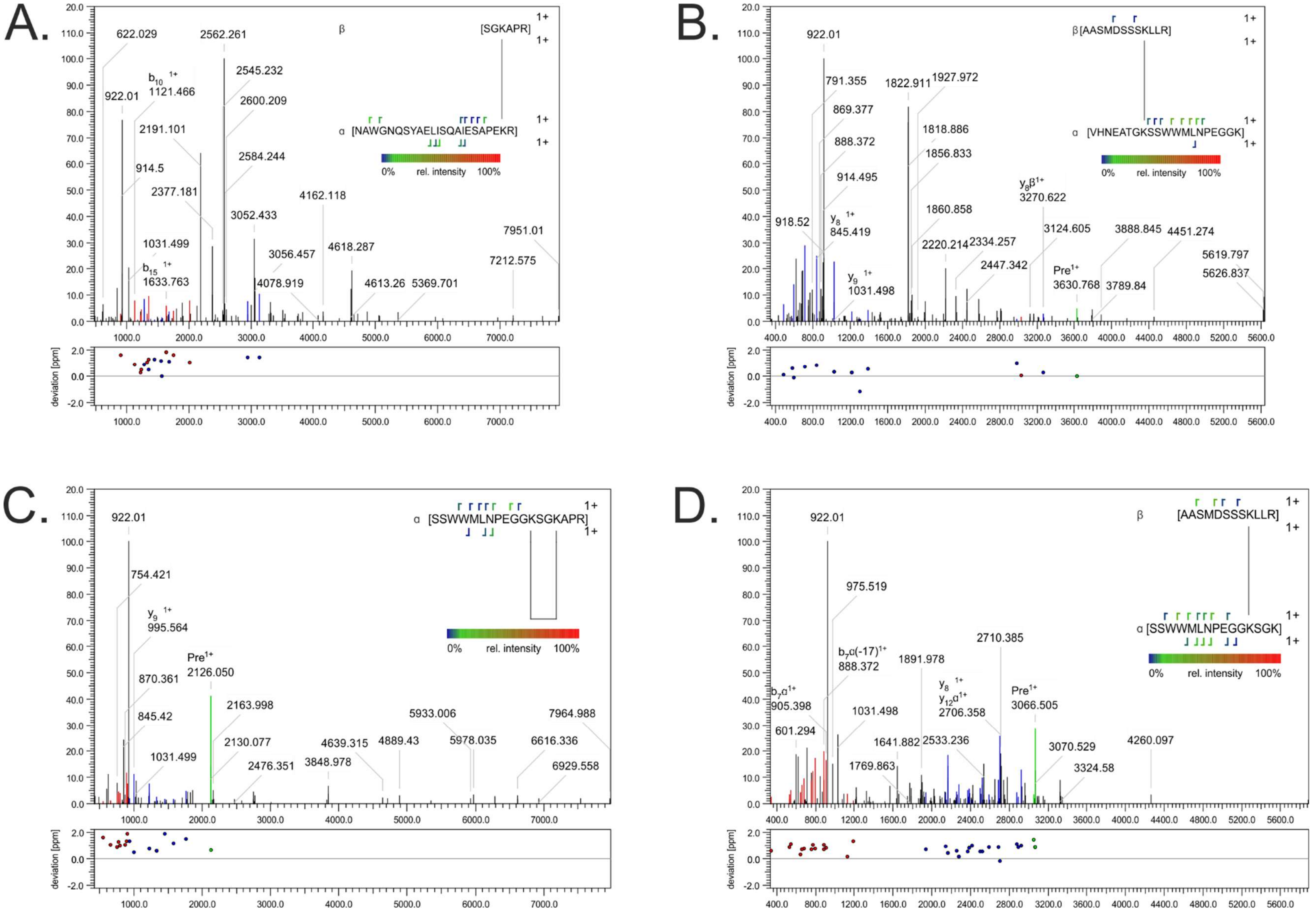

Figure S25: Mass spectra of cross-links dissociation products (16/17). Each spectrum represents a single identified peptide-peptide cross-linked by DSS [di(N-succinimidyl) suberate] (represented by line connecting two cross-linked residues): **A.** K_{166} - K_{185}; **B.** K_{170} - K_{199}; **C.** K_{182} - K_{185}; **D.** K_{182} - K_{199}. Fragmentation sites are marked along the amino acids sequence of each peptide. Resulting fragments are highlighted and designated in each spectrum.

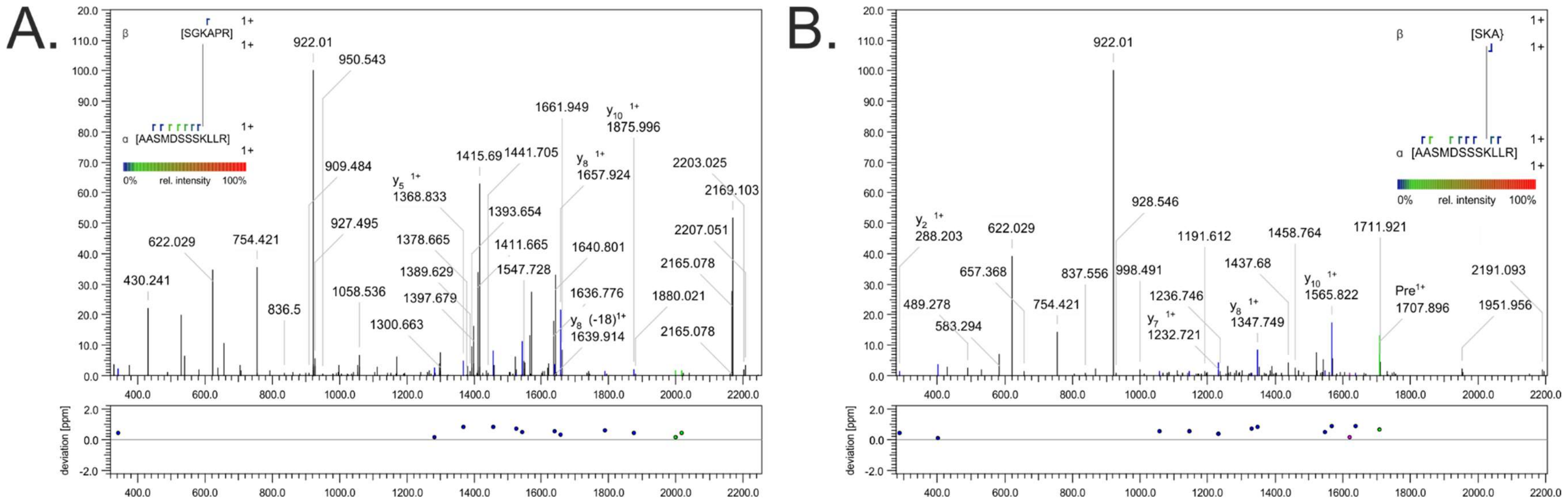

Figure S26: Mass spectra of cross-links dissociation products (17/17). Each spectrum represents a single identified peptide-peptide cross-linked by DSS [di(N-succinimidyl) suberate] (represented by line connecting two cross-linked residues): **A.** K_{185} - K_{199}; **B.** K_{199} - K_{206}. Fragmentation sites are marked along the amino acids sequence of each peptide. Resulting fragments are highlighted and designated in each spectrum.

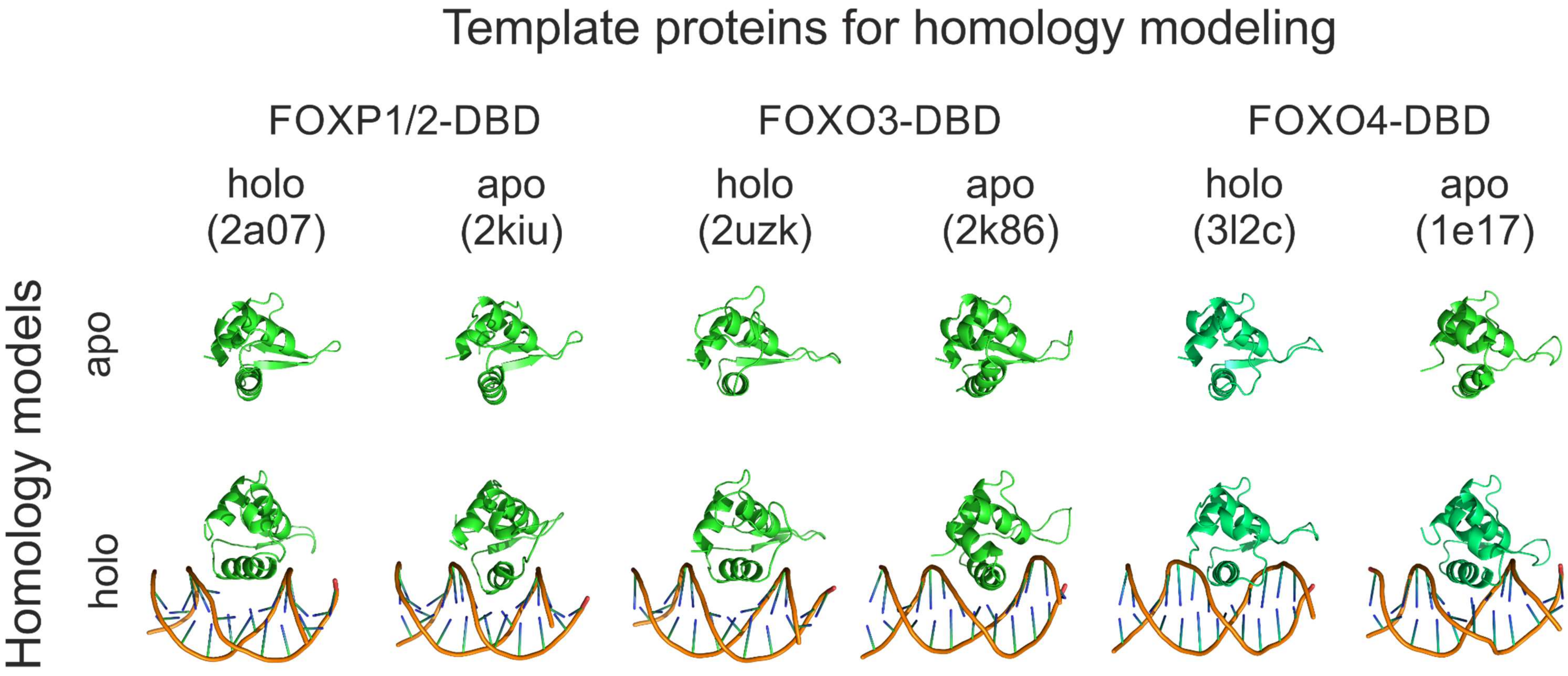

Figure S27: Protein-DNA docking. Homology models (green) of apo and holo form of FOXO4-DBD based on available sequentially related three-dimensional structures [FOXO4-DBD (100% sequence identity (s.i.), FOXO3-DBD (83% s. i.), and FOXP1/2-DBD (51% s. i.]. DBE substrate (partially orange) were further docked into the holo form models of FOXO4-DBD according to the HDX experiments to create full holo form model of FOXO4-DBD•DBE complex.

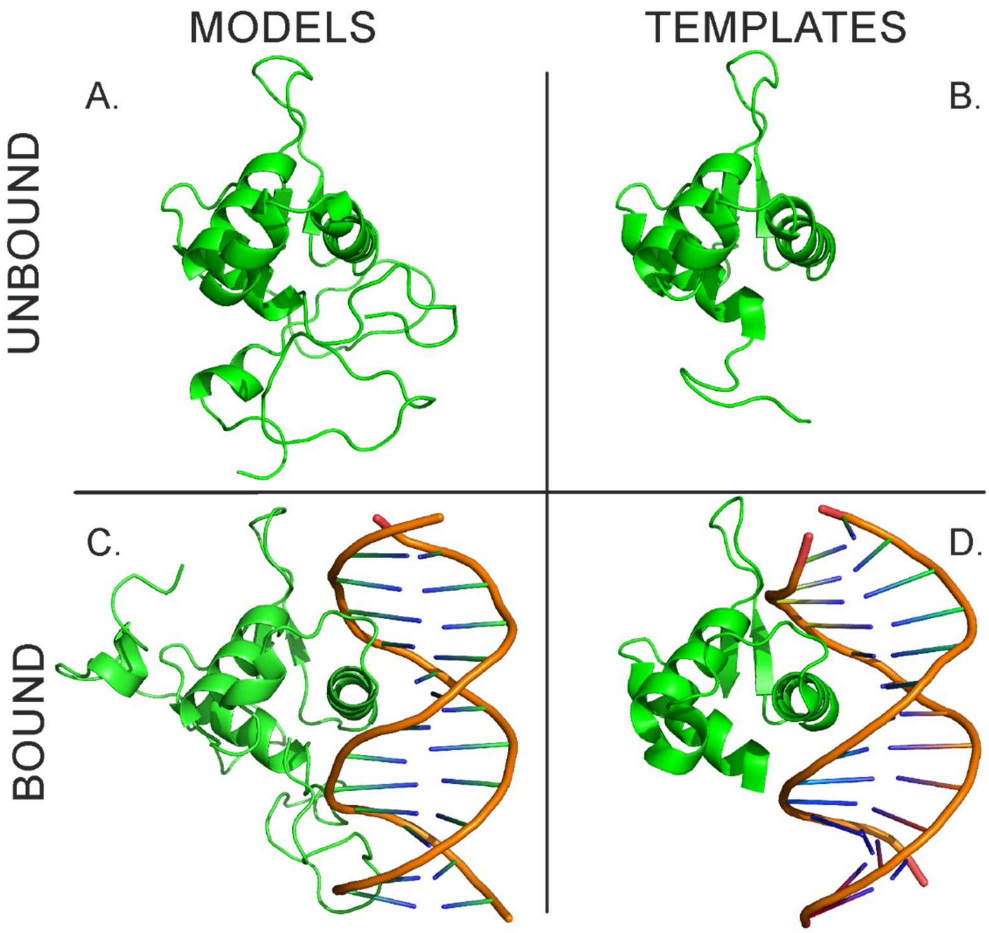

Figure S28: Comparison between models of full-fledged FOXO4-DBD in either free (**A.**) or DBE-bound (**C.**) form with the corresponding high-resolution structures 1E17 (**B.**) and 3L2C (**D.**), which were used as templates in the homology modelling operations.

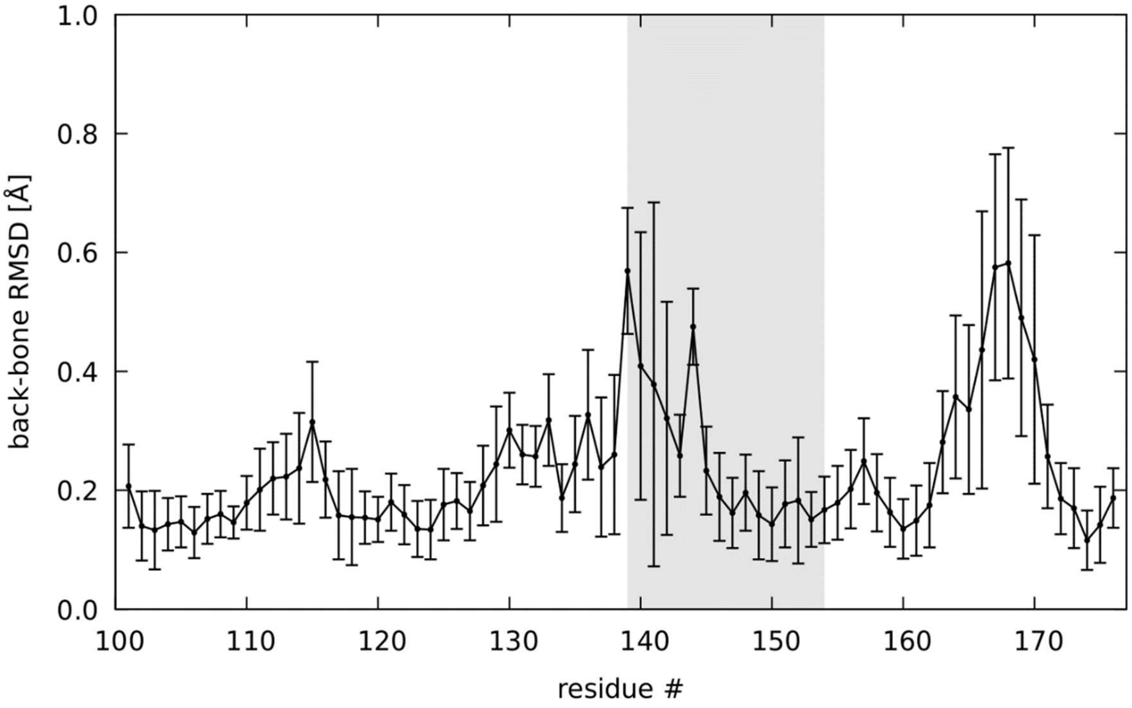

Figure S29: Average RMSD between the coordinates of backbone heavy atoms in our unbound FOXO4-DBD model and the corresponding 1E17 high-resolution template. Error bars represent the standard deviation produced

by the various structures in the model ensemble. The greyed area corresponds to the H3 helix region, which experienced the most significant variations upon binding.

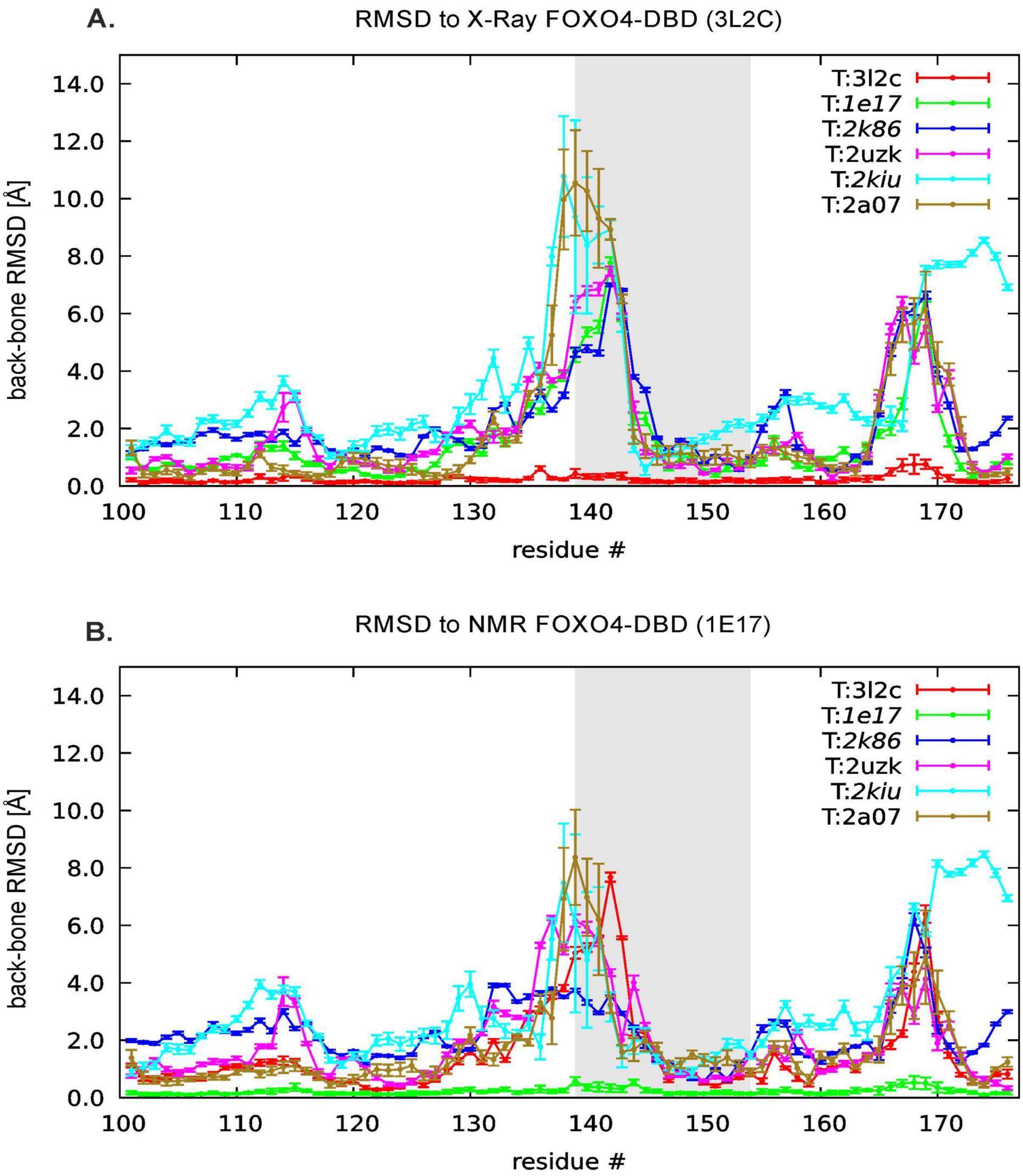

Figure S30: Average RMSD between the coordinates of backbone heavy atoms in six created unbound FOXO4-DBD models based on different FOX structures related to FOXO4 and the 3L2C (**A.**) or 1E17 (**B.**) high-resolution template. Error bars represent the standard deviation produced by the various structures in the model ensemble. The greyed area corresponds to the H3 helix region, which experienced the most significant variations upon binding.

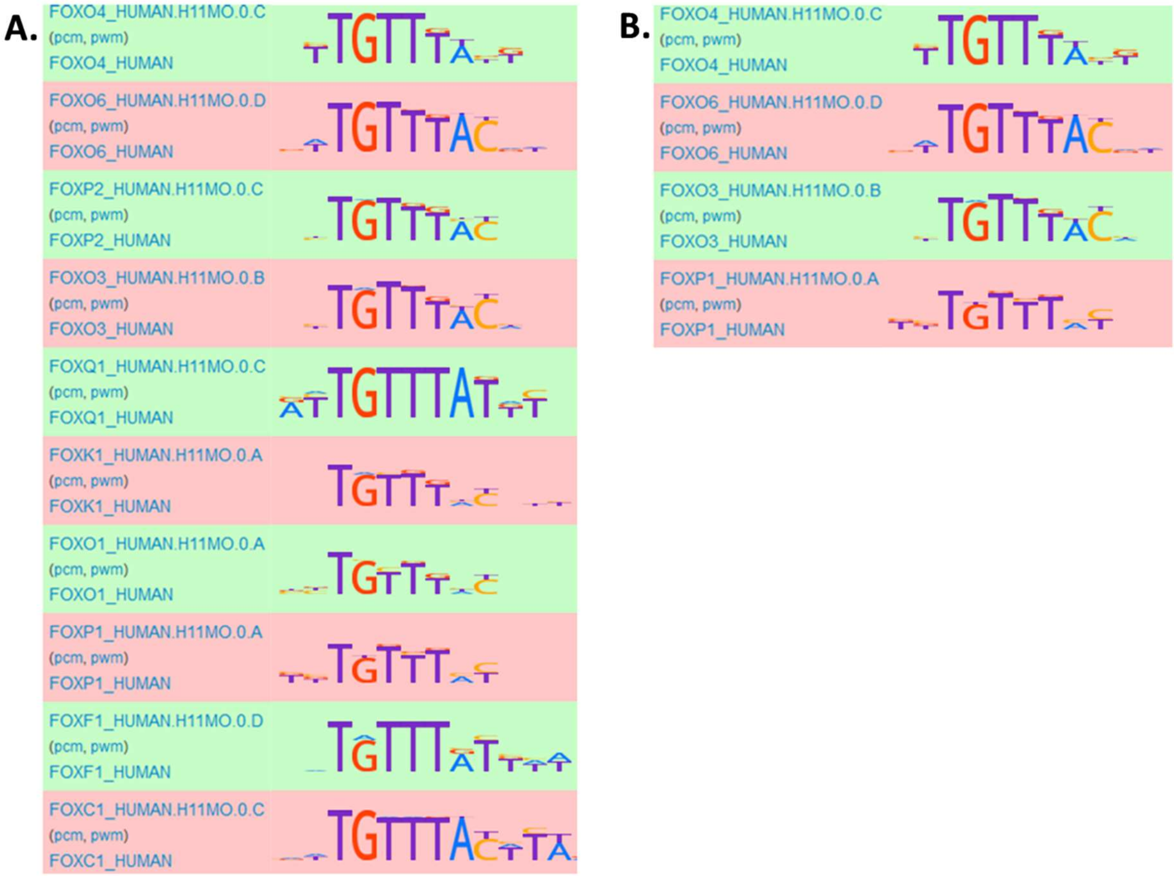

Figure S31: Putative FOXO4 consensus sequences. **A.** The TGTTT consensus sequence is highly conserved in the binding models of numerous other FOX factors. **B.** The FOXO4 consensus sequence is closely related to those of FOXO6, FOXO3 and FOXP1, as determined by comparison of all Position Count Matrix (PCM) models in the HOCOMOCO-11. These data were obtained by using MACRO-APE software as described above.

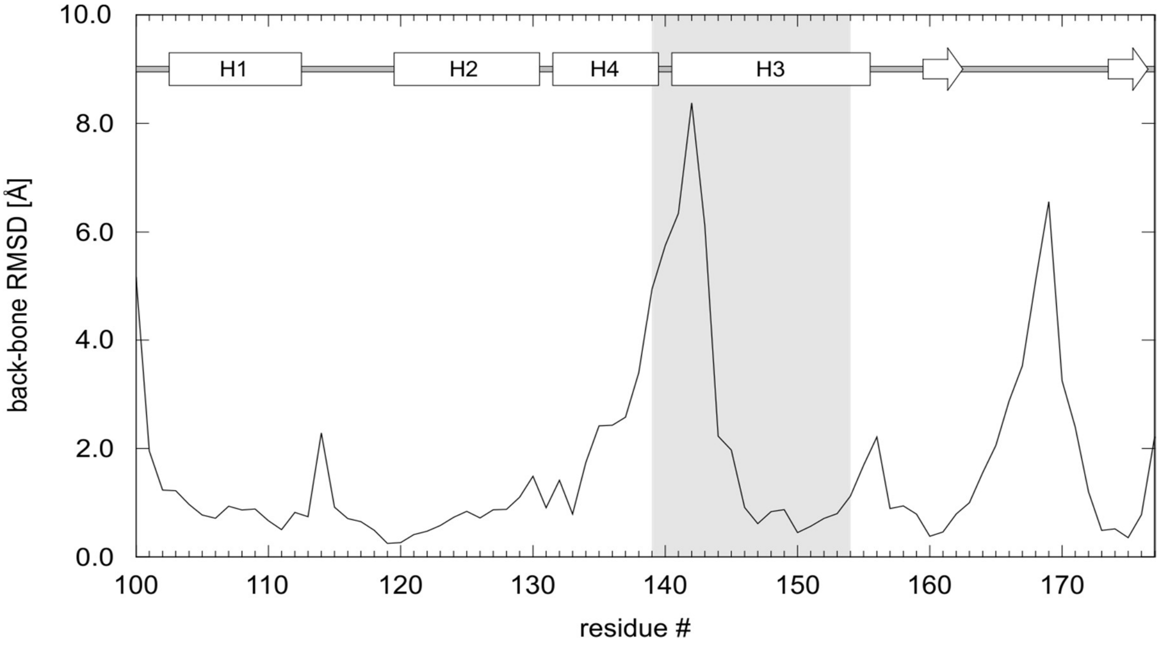

Figure S32: Average RMSD between the coordinates of backbone heavy atoms in our unbound FOXO4-DBD and bound FOXO4-DBD•DBE complex. The greyed area corresponds to the H3 helix region, which experienced the most significant variations upon binding. However, the plot highlights additional regions located away from the contact interface, which were significantly affected by the interaction with DBE.

GSSHHHHHHSSGLVPRGSHMLEDPG_{82}AVTGPRKGGSRRNAWGNQSYAELISQA
IESAPEKRLTLAQIYEWMVRTVPYFKDKGDSNSSAGWKNSIRHNLSLHSKFIKV
HNEATGKSSWWMLNPEGGKSGKAPRRRAASMDSSSKLLRGRSKA_{207}

Figure S33: Protein sequence encoded by the express vector pET-15b. Cleavage site marked as a red part of polyhistidine tag, which is marked in black. Blue part represents the wild-type FOXO4-DBD.

3. Tables

	Cross-linked residues			Cross-linker	Max. theor. dist. [Å]	Bound model dist. dif. [Å]	Unbound model dist. dif. [Å]
Cross-links used for building of model of bound form	N-term	-	K89	DSG	14.1	-2.9	-0.2
	N-term	-	K116		14.1	2.4	2.4
	N-term	-	K135		14.1	1.5	2.2
	N-term	-	K137		14.1	1.7	1.9
	N-term	-	K159		14.1	-1.9	-2.2
	N-term	-	K199		14.1	3.1	2.8
	K89	-	K182		20.5	-10.4	-1.4
	K116	-	K182		20.5	-6.5	-3.6
	K135	-	K137		20.5	-14.9	-15.3
	K159	-	K182		20.5	-14.5	-8.0
	K159	-	K199		20.5	0.2	-11.8
	K162	-	K170		20.5	-7.3	-7.9
	K162	-	K182		20.5	-6.5	-0.5
	K162	-	K199		20.5	-1.4	-7.4
	K162	-	K206		20.5	2.4	-5.4
	K170	-	K199		20.5	-4.7	3.0
	K182	-	K185		20.5	-13.1	-12.1
	K182	-	K199		20.5	2.8	-4.8
	K185	-	K199		20.5	-0.5	-2.2
	N-term	-	K162	DSS	17.8	-2.4	-2.8
	N-term	-	K170		17.8	2.4	3.0
	N-term	-	K182		17.8	-0.4	0.4
	N-term	-	K185		17.8	2.4	0.2
	N-term	-	K206		17.8	3.0	2.4
	K116	-	K159		24.2	-11.2	-10.0
	K116	-	K162		24.2	-11.8	-11.0
	K135	-	K147		24.2	-11.1	-11.9
	K162	-	S172		20.2	-13.8	-13.9
	K199	-	K206		24.2	-7.8	-13.2
	S171, S172 or M175	-	DBE_R	tPt	5.5	2.8	2.9
	S75, H76, M77 or T85	-	DBE_R		5.5	-0.5	-0.9
	H164 or T168	-	DBE_F		5.5	4.3	5.6
	S171, S172 or M175	-	DBE_F		5.5	3.3	3.8
	S149, H152, S155, H157 or S158	-	DBE_F		5.5	-2.4	-2.5
	S75, H76, M77 or T85	-	DBE_F		5.5	-2.7	-1.5
	S75, H76, M77 or T85	-	THY2 (DBE_F)		5.5	2.5	2.4

Table S1 (1/2): Deviations between theoretical and back-calculated distances of cross-linked residues in unbound and bound forms obtained from our structural models.

	Cross-linked residues	Cross-linker	Max. theor. dist. [Å]	Bound model dist. dif. [Å]	Unbound model dist. dif. [Å]
Cross-links used for building of model of unbound form	N-term - K89	DSG	14.1	-2.4	-1.9
	N-term - K137		14.1	2.4	2.0
	N-term - K147		14.1	-3.7	-4.2
	N-term - K185		14.1	1.7	2.5
	N-term - K199		14.1	-2.3	-2.0
	K89 - K135		20.5	-3.6	-1.7
	K89 - K199		20.5	-2.4	-1.5
	K116 - K182		20.5	-1.4	-1.9
	K116 - K185		20.5	-0.5	0.1
	K116 - K199		20.5	1.5	1.0
	K135 - K137		20.5	-15.0	-15.0
	K135 - K147		20.5	-7.0	-7.4
	K135 - K199		20.5	1.2	1.0
	K137 - K147		20.5	-10.6	-9.3
	K147 - K170		20.5	-2.9	-1.0
	K159 - K199		20.5	-4.2	-3.7
	K162 - K170		20.5	-7.6	-7.0
	K182 - K185		20.5	-11.4	-11.8
	K185 - K199		20.5	-4.1	-4.3
	K199 - K206		20.5	-7.4	-7.2
	N-term - K206	DSS	17.8	0.3	0.2
	K89 - K116		24.2	1.8	0.4
	K147 - K162		24.2	-7.2	-5.8
	K162 - S172		20.2	-14.1	-13.3

Table S1 (2/2): Deviations between theoretical and back-calculated distances of cross-linked residues in unbound and bound forms obtained from our structural models.

Cross-linked residues	DSG		DSS	
	Unbound (d0) [%]	Bound (d4) [%]	Unbound (d0) [%]	Bound (d4) [%]
N-term - K89	55.7 (0.3)	44.3 (0.3)	37.5 (1.7)	62.5 (1.7)
N-term - **K116**	33.1 (1.4)	66.9 (1.4)	29.3 (0.7)	70.7 (0.7)
N-term - **K135**	24.1 (2.5)	75.9 (2.5)	19.0 (2.5)	81.0 (2.5)
N-term - **K137**	48.5 (5.6)	51.5 (5.6)	5.6 (1.9)	94.4 (1.9)
N-term - **K147**	92.8 (0.4)	7.2 (0.4)	72.7 (3.2)	27.3 (3.2)
N-term - **K159**	27.4 (6.6)	72.6 (6.6)	2.9 (0.4)	97.1 (0.4)
N-term - **K162**	-	-	32.6 (4.8)	67.4 (4.8)
N-term - **K170**	-	-	2.6 (1.1)	97.4 (1.1)
N-term - K182	-	-	4.7 (0.8)	95.3 (0.8)
N-term - K185	70.6 (3.4)	29.4 (3.4)	26.7 (2.0)	73.3 (2.0)
N-term - K199	62.5 (0.6)	37.5 (0.6)	38.2 (1.1)	61.8 (1.1)
N-term - K206	-	-	57.7 (0.7)	42.3 (0.7)
K89 - **K116**	-	-	78.5 (2.9)	21.5 (2.9)
K89 - **K135**	72.4 (2.1)	27.6 (2.1)	-	-
K89 - K182	15.7 (2.1)	84.3 (2.1)	17.6 (3.0)	82.4 (3.0)
K89 - K199	72.4 (3.3)	27.6 (3.3)	-	-
**K116** - **K159**	-	-	28.3 (2.2)	71.7 (2.2)
**K116** - **K162**	-	-	14.9 (3.2)	85.1 (3.2)
**K116** - K182	37.4 (4.0)	62.6 (4.0)	37.0 (5.2)	63.0 (5.2)
**K116** - K185	86.1 (1.9)	13.9 (1.9)	68.5 (1.7)	31.5 (1.7)
**K116** - K199	86.9 (3.7)	13.1 (3.7)	83.2 (1.6)	16.8 (1.6)
**K135** - **K137**	40.7 (5.1)	59.3 (5.1)	31.7 (2.4)	68.3 (2.4)
**K135** - **K147**	79.3 (1.9)	20.7 (1.9)	61.5 (1.5)	38.5 (1.5)
**K135** - K199	79.4 (3.8)	20.6 (3.8)	-	-
**K137** - **K147**	85.2 (2.0)	14.8 (2.0)	-	-
**K147** - **K162**	-	-	100 (0.0)	0 (0.0)
**K147** - **K170**	95.9 (3.6)	4.1 (3.6)	100.0 (0.0)	0 (0.0)
**K159** - K182	13.7 (4.4)	86.3 (4.4)	13.3 (1.8)	86.7 (1.8)
**K159** - K199	57.6 (6.2)	42.4 (6.2)	-	-
**K162** - **K170**	36.1 (5.1)	63.9 (5.1)	37.6 (4.5)	62.4 (4.5)
**K162** - **S172**	-	-	35.0 (2.3)	65.0 (2.3)
**K162** - K182	9.9 (2.9)	90.9 (2.9)	9.7 (3.5)	90.3 (3.5)
**K162** - K199	11.6 (2.4)	88.4 (2.4)	5.7 (2.7)	94.3 (2.7)
**K162** - K206	11.8 (2.6)	88.2 (2.4)	3.3 (0.5)	96.7 (0.5)
**K170** - K199	26.3 (5.1)	73.7 (5.1)	22.6 (3.8)	77.4 (3.8)
K182 - K185	53.0 (3.1)	47.0 (3.1)	43.2 (0.8)	56.8 (0.8)
K182 - K199	29.6 (4.9)	70.4 (4.9)	7.9 (4.8)	92.1 (4.8)
K185 - K199	65.0 (20.1)	35.0 (2.1)	65.7 (1.5)	34.3 (1.5)
K199 - K206	69.5 (3.2)	30.5 (3.2)	51.0 (0.7)	49.0 (0.7)

Table S2: Quantitative protein-protein cross-linking of unbound FOXO4-DBD and bound FOXO4-DBD•DBE (complex). Cross-linked residues in bold were present also in the high-resolution template employed for homology modelling. Cross-linking experiments were done in triplicate. The percentages presented in this table are average

values. The standard deviation is given in brackets. Abbreviations: DSG - disuccinimidyl glutarate, DSS - disuccinimidyl suberate. Note that the G_{74} - P_{87} region of our construct was introduced by the vector used for recombinant production, and thus was not part of the wildtype FOXO4 sequence (see **Supporting Information Figure S14**).
